# Homozygosity Accumulated Through Genetic Drift and Inbreeding Underpins High Mutation Load in an Endangered Beluga Whale Population

**DOI:** 10.1111/mec.70521

**Published:** 2026-08-21

**Authors:** R. W. Orton, C. Müller, C. J. Garroway, S. H. Ferguson, R. Michaud, T. R. Frasier

**Affiliations:** ^1^ Department of Biology Saint Mary's University Halifax Nova Scotia Canada; ^2^ Department of Biological Sciences University of Manitoba Winnipeg Manitoba Canada; ^3^ Department of Fisheries and Oceans Freshwater Institute Winnipeg Manitoba Canada; ^4^ Group for Research and Education of Marine Mammals Taduoussac Quebec Canada

**Keywords:** genetic drift, genetic purging, isolation, mutation load, population size

## Abstract

Isolated populations are susceptible to decline through the accumulation of deleterious mutations and inbreeding depression. As a consequence of habitat alteration, isolated populations are also expected to become increasingly prevalent. Still, some isolated populations have evaded decline through the purging of deleterious mutations. Expanding our knowledge of the scenarios under which purging occurs could therefore improve conservation efforts. Although theoretical and captive studies suggest that genetic purging hinges on a population's demographic history, the factors that shape the probability of purging in natural populations are less resolved. Here, we infer demographic parameters and quantify genetic variation in four Canadian populations of beluga whale to gain insight into purging. In particular, we explore the dynamics of the Saint Lawrence Estuary (SLE), a population that shows signatures of genetic erosion. We find that the SLE has been isolated for hundreds of generations and has accumulated a high mutation load in the absence of purging. We also find evidence of inbreeding from throughout the history of the SLE, excluding the most recent generations. Among deleterious alleles in the SLE, we identify eight clusters unique to neonate mortality—four of which are associated with cellular responses to inorganic substances. Given industrial pollution in the SLE, this finding indicates a relationship between environment, genetic variation and neonate mortality. Our results suggest that the likelihood of purging in natural populations may be reduced when mutation load accumulates via sustained isolation, highlighting the importance of demographic history in shaping conservation risk.

## 1 Introduction

As anthropogenic activities continue to deplete global biodiversity, the ability to quantify and interpret the factors that underpin population viability will become increasingly valuable. Conservation efforts tend to be prioritized towards small and isolated populations, in part because those populations are often characterized by high rates of genetic drift and inbreeding (Frankel and Soulé 1981; Frankham 2018) that can lead to the accumulation of deleterious alleles (mutation load [Muller 1950]) and inbreeding depression (Darwin 1877). In response to expanding habitat loss and fragmentation, isolated populations are expected to become more prevalent, and increasingly small until extinction (Fahrig 1997; Diaz et al. 2014). However, a growing body of theoretical (e.g., Pérez‐Pereira et al. 2022; Xie et al. 2022) and empirical (e.g., Ochoa and Gibbs 2021; Crossman, Fontaine, and Frasier 2024; Crossman, Hamilton, et al. 2024) research indicates that small population size and isolation do not necessarily doom populations to extinction. Studies of island foxes ( *Urocyon littoralis* ) and the Ethiopian wolf ( *Canis simensis* ), for example, reveal no clear genomic signals of inbreeding depression despite long‐term small population size in isolation (Robinson et al. 2018; Mooney et al. 2023). Improving our understanding of the evolutionary dynamics of small and isolated populations will therefore enable us to better conserve biodiversity given current global declines.

An emerging consensus across studies of evolution is that small and isolated populations might be able to evade extinction through a mechanism known as genetic purging (Hedrick 1994; Hedrick and Garcia‐Dorado 2016; Van Der Valk et al. 2021). This mechanism occurs in small populations when purifying selection targets (partially) recessive deleterious alleles expressed in homozygous genotypes. The process of genetic purging is most often considered a potential outcome of inbreeding (as in Hedrick and Garcia‐Dorado 2016); however, it may also occur as a result of genetic drift (Glémin 2003). Regardless of cause, the restorative effect of genetic purging occurs through the reduction of frequencies of deleterious alleles segregating within a population. This is illustrated in the history of the Northern elephant seal ( *Mirounga angustirostris* ), where the species' recovery coincides with the purging of the *realized* mutation load (deleterious homozygotes) that accumulated during a severe population bottleneck initiated by overexploitation (Hoffman et al. 2024). Still, genetic purging is not universal in nature (as reviewed by Byers and Waller 1999; Taylor et al. 2024).

The probability that genetic purging occurs in declining populations is so far thought to hinge on the rate of population contraction and the size of the ancestral population (Caballero et al. 2017; López‐Cortegano et al. 2021), because the efficacy of natural selection relative to the strength of genetic drift decays as population size decreases (Kimura et al. 1963). Abrupt population contractions can lead to increases in the frequency and potential fixation of recessive deleterious alleles at some sites through genetic drift that outpaces purifying selection (Van Der Valk et al. 2021). Moreover, effective genetic purging can be impeded in bottlenecked populations that are descended from large ancestral populations, because larger populations tend to shelter elevated frequencies of deleterious recessive alleles within high genome‐wide heterozygosity (as reviewed by Charlesworth and Willis 2009). A high *potential* mutation load (deleterious heterozygotes) hence increases the chances of a high realized mutation load following a population contraction (Kyriazis et al. 2021; as reviewed by Robinson et al. 2023). Nevertheless, our understanding of the links between demographic parameters and genetic purging is largely derived from theoretical simulations (Lynch et al. 1995; Caballero et al. 2017) and laboratory experiments (López‐Cortegano et al. 2021). Although the utility of these studies is invaluable, their conclusions may not always reflect the dynamics of natural populations. In particular, many of these studies have either assumed or prompted inbreeding. Although inbreeding avoidance in whales is not well understood (e.g., Frasier et al. 2013), inbreeding avoidance in nature has been documented (as reviewed by Pusey and Wolf 1996). Across the empirical studies to date, the associations between demographic history and genetic purging are varied (Grossen et al. 2020; Xie et al. 2022; Taylor et al. 2024). To more thoroughly comprehend genetic purging in natural populations, additional empirical data are required.

Combining inferences of demographic history and mutation load across target and closely related populations in nature contributes to our understanding of genetic purging, enables broad predictions of extinction probabilities, and may form the basis of genetic monitoring programs. This approach can also be leveraged to contextually highlight the impacts of anthropogenic activity on population dynamics, because the extent of suitable habitat and connectivity between populations influences population size and gene flow (as reviewed by Lino et al. 2019; Pinto et al. 2024). The Pleistocene glaciations, for example, led to cycles of population isolation (in glacial refugia) and secondary contact which shaped genetic diversity across an array of taxa, such as red rattlesnakes ( *Crotalus ruber* ) (Harrington et al. 2018) and Fennoscandian moose ( *Alces alces* ) (Kangas et al. 2015). As such, the distribution of allele frequencies by way of demographic change related to landscape alteration could impact the probability of future genetic purging. Currently, the reductions of sea ice levels due to global change are altering the extent of suitable habitat and connectivity across the Arctic at a pace that is four times faster than the global rate (Rantanen et al. 2022). These changes could affect the population dynamics of Arctic and subarctic species, such as the beluga whale (*Delphinapterus leucus*).

In Eastern Canada, there are seven ‘Designatable Units’ (DUs) of beluga identified by The Committee on the Status of Endangered Wildlife in Canada (COSEWIC 2016); however, recent genetic studies have identified six genetic clusters (Montana et al. 2024; Müller et al. 2026). Although the species is listed as ‘Least Concern’ at the global scale by the IUCN (Lowry et al. 2017), several DUs, such as that of the Saint Lawrence Estuary (SLE), are listed as endangered in Canada (COSEWIC 2020). The SLE has an estimated census size of fewer than 2000 individuals (Hobbs et al. 2019; DFO 2023) and suffers from high rates of disease, cancer and neonate mortality (Lair et al. 2016). Although vessel traffic and industrial contamination in the Saint Lawrence Estuary threaten the health of the belugas (Williams et al. 2021), many of the maladies presented by the SLE, such as high rates of neonate mortality, also mirror certain symptoms of inbreeding depression (as reviewed by Charlesworth and Willis 2009). The SLE is at the southern limit of the species' range and is not thought to overlap with the distribution of other Canadian beluga (Bailleul et al. 2012), effectively isolating the DU. Due to its relatively small population size and apparent isolation, one hypothesis is that high mutation load and inbreeding depression may be impacting the population dynamics of the SLE.

To explore the relationships between mutation load, genetic purging and demographic history in beluga, we resequenced a total of 40 whole genomes from across Eastern Canada. In particular, we resequenced genomes of 10 adult belugas from the Saint Lawrence Estuary, as well as from three additional sites for comparison. These included the Hudson Strait near Nunavik (NVK), the Eastern Hudson Bay near the Belcher Islands (BIS) and Cunningham Inlet in the Eastern High Arctic (EHA). Utilizing these genomic data, we inferred demographic history and estimated genetic diversity (and differentiation), inbreeding, and the mutation load of each group. To more specifically characterize the population dynamics of the endangered SLE, we also resequenced whole genomes from eight belugas that died as neonates in the Saint Lawrence Estuary. The landscapes of homozygosity and mutation load across those genomes were then compared to those of the adults from the SLE to identify key mutations that could be associated with its current conservation status.

## 2 Methods

### 2.1 Sample Selection and Sequencing

**FIGURE 1:**
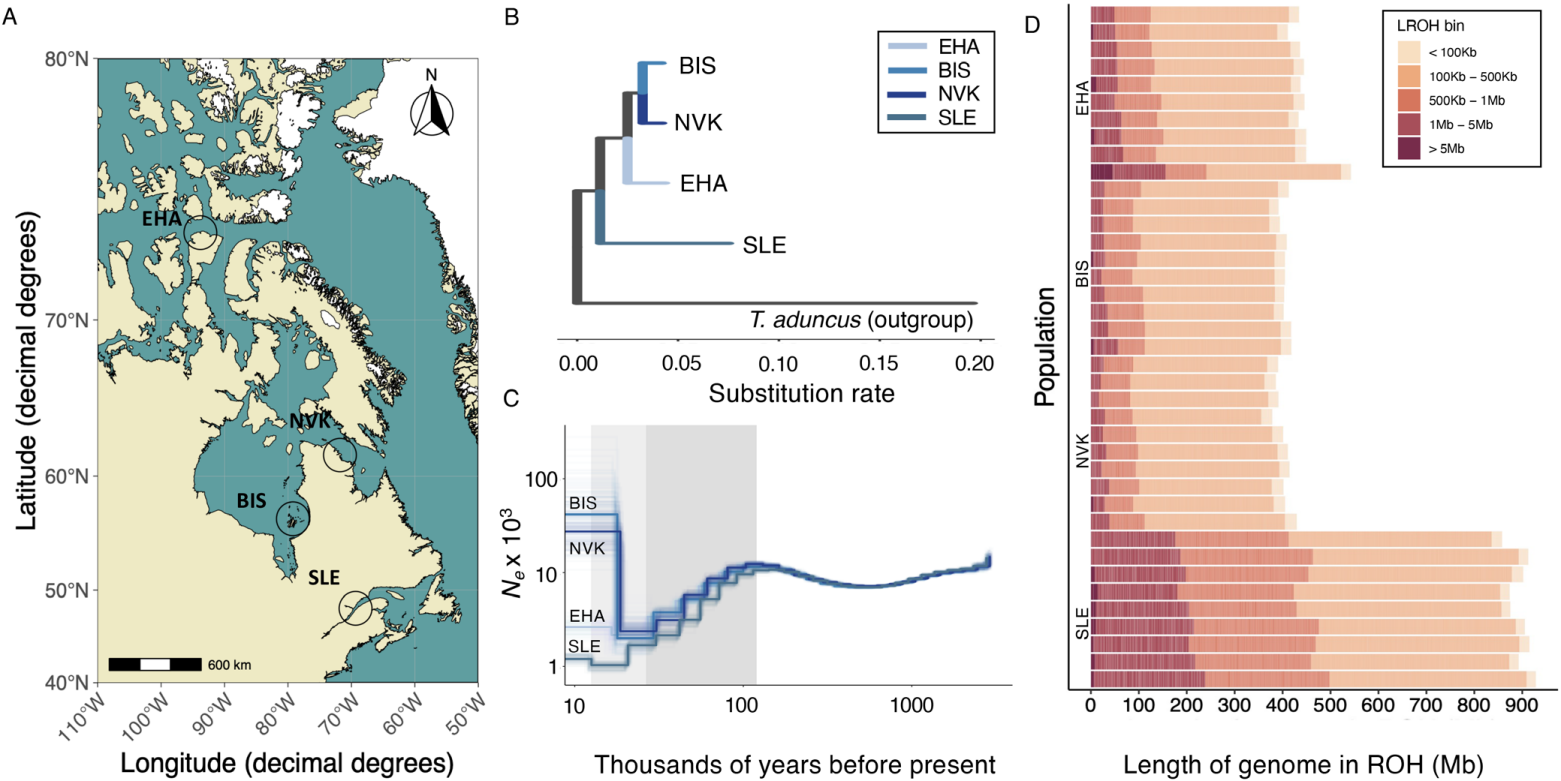
Estimates of genetic differentiation and demographic history among four Eastern Canadian beluga populations. (A) The summering locations from where genomes were sampled: Saint Lawrence Estuary (SLE), Nunavik (NVK), the Belcher Islands (BIS) and the Eastern High Arctic (EHA). Longitude and Latitude are labelled along the *X* and *Y* axes, respectively. Current glaciers are shown in white. (B) The fully supported cladogram of the four beluga populations generated with BEAST2. The Indo‐Pacific bottlenose dolphin was used as the outgroup. (C) Changes in the effective population size (*N*_e_) across the last three million years for each of the four beluga populations inferred with 100 iterations of PSMC. We used a generation time of 32 years and a mutation rate of 1.65 × 10^−8^. The dark grey shaded area denotes approximately the Last Glacial Period, and the lighter shaded area shows approximately the Last Glacial Maximum. (D) Runs of homozygosity (ROH) of five different bin sizes (shown in legend) for each individual within each population. For (B, C), population is consistently denoted by the colour shown in the legend of (B).

We sampled genomes from 40 adult belugas that were evenly distributed across the four Canadian beluga populations (*N* = 10 each). In addition, we sampled genomes from eight necropsied neonate mortalities from the SLE. Necropsies were performed following their field collections between 1995 and 2020. These individuals were each thought to have been the offspring of adult residents of the Saint Lawrence Estuary (Table S1). For the 18 samples (adults and neonates) from the SLE and the 10 from NVK (Figure 1a), we sent 1–5 μg of genomic DNA to the McGill Applied Genomics Innovation Core (Montreal, Quebec, Canada) where samples were PCR‐free library prepped using a NxSeq AmpFREE kit (Lucigen, Wisconsin, USA). For sequencing, libraries were pooled across three Illumina NovaSeq 6000 S4 lanes using 150‐bp paired‐end reads. For the 10 samples from BIS and the from the EHA were sequenced at the Centre for Applied Genomics of the Hospital for Sick Children in Toronto. The LSK‐110 kit was used for library preparation (Müller et al. 2026). These reads were pooled across four Illumina NovaSeq 6000 S4 lanes. All raw Beluga reads were demultiplexed at the respective core facilities. To polarize ancestral and derived alleles, we also downloaded three harbour porpoise ( *Phocoena phocoena* ), three Indo‐Pacific bottlenose dolphin ( *Tursiops aduncus* ) and three finless porpoise ( *Neophocaena asiaeorientalis asiaeorientalis* ) genomes using Sequence Read Archive Toolkit (https://trace.ncbi.nlm.nih.gov/Traces/sra/sra.cgi?view=software) *prefetch* and *faster‐q dump*. These genomes were deposited to NCBI by Celemín et al. (2023), Vijay et al. (2018) and Zhou et al. (2018), respectively.

### 2.2 Processing Reads and Variant Calling

We trimmed and filtered all reads using Trimmomatic v0.39 (Bolger et al. 2014). Reads with Phred scores below 30, a minimum length of 32, an average quality below 32, or leading or trailing ends with scores below 20 were immediately filtered. We then used BWA v0.7.12 *mem* (Li and Durbin 2009) to map reads to the 21 autosomes of the beluga whale reference, ASM228892v2_HiC, found on DNA Zoo (https://www.dnazoo.org/assemblies/delphinapterus_leucas). This reference was based upon the draft assembly from (Jones et al. 2017) using the Hi‐C approach (Dudchenko et al. 2017). We opted to use this version of the beluga whale reference because of its chromosome‐level assembly and scaffold N‐50 of 107 Mb. In comparison, ASM228892v3, the scaffold‐level assembly updated from Jones et al. (2017), has a scaffold N‐50 of 31.2 Mb. The longer scaffolds allowed us to make inter‐chromosomal comparisons that would be reflective of the beluga whale karyotype. Once reads were mapped, they were sorted and indexed using samtools v1.17 (Li and Durbin 2009). We marked duplicates across merged reads with Picard (http://broadinstitute.github.io/picard/) and GATK (Van der Auwera and O'Connor 2020). Repetitive regions were masked using bcftools *view* and the reference assembly repeats bed file. We called variants using bcftools v1.18 (Li and Durbin 2009) *mpileup* and *call* commands and removed multiallelic sites and indels using *view*. This pipeline yielded a ‘base’ gVCF file of 79,637,856 sites with 8,279,781 variant sites across 57 samples (including the nine outgroup samples). Counts and mapping statistics were generated with the bcftools *counts* plugin and samtools *stats*.

To polarize the ancestral allele at each site, we subset a new gVCF with only the nine outgroup samples. Site level statistics, including read depth, alternate allele frequency and allele counts were regenerated using bcftools *+fill‐tags*. Using bcftools, we then filtered this gVCF file by removing sites where any mapped sample had a read depth less than 11 or where the site had an average mapping quality below 40. The ancestral allele at each retained site was then considered the same as the beluga reference allele unless the alternate allele segregated across outgroup samples at or above a frequency of 0.70. For any sites missing outgroup data, we assigned the reference allele as the ancestral allele using custom scripts in bash. We annotated the ancestral allele at each site in the ‘base’ gVCF file with all 57 samples using the bcftools *annotate* command. Although we acknowledge that this method of polarization could relax constraints for inferring derived alleles, we also note that it may be important to consider that lower impact deleterious mutations can segregate at low frequencies in demographically stable populations (Agrawal and Whitlock 2012). Here, we did not want to exclude such mutations from our analyses of mutation load. Our method of polarization was also similar to other recent studies of mutation load, including Grossen et al. (2020), Smeds and Ellegren (2023) and Nigenda‐Morales et al. (2023).

Importantly, we parsed two different gVCF files from the polarized base gVCF described above. First, we parsed a gVCF file that included only the 40 adult belugas in this study. Note that because neonate mortalities will not have contributed alleles to the current population, we excluded them from comparative analyses across populations. We regenerated new statistics for each site using bcftools *+fill‐tags* and refiltered sites using the same criteria as stated above. We removed any sites that had missing data, and we retained invariant sites in this ‘population‐level’ gVCF as certain analyses require invariant sites (*see below*). This yielded a gVCF file that contained 72,070,279 total sites, 779,009 of which were variant. Second, we used the 10 adults and eight neonate mortalities from the SLE to assess population dynamics in the SLE. For analyses of population dynamics within the SLE, we removed all invariant sites and any sites with missing data from the ‘SLE‐specific’ gVCF file. We also recalled site‐level statistics using bcftools *+fill‐tags* and refiltered the file using the same criteria as above. These steps yielded a VCF of 1,856,900 variant sites. Note that the lower number of variant sites in the population‐level file compared to the SLE‐specific file is due to differences in mapping statistics, where the probability of missing data at any one site increases as individuals are added. Variation in the number of segregating sites for each analysis likely accounts for the differences in estimates of homozygosity and mutation load for the adults. We still opted to use this approach because counts of deleterious mutations can be strongly biassed if missing data are unevenly distributed across populations or groups (Agrawal and Whitlock 2012; Dehasque et al. 2024). We also note that the overall patterns of homozygosity and mutation load for the adults are similar for both analyses despite a differing number of variant sites.

### 2.3 Genetic Differentiation and Diversity

We used Admixture (Alexander and Lange 2011) to gain initial insight as to stratification across the four Canadian beluga populations. To remove the potential for linkage that could influence our results, we ran Admixture across a dataset that was pruned of any variant sites correlated above an *r* ^2^ value of 0.6 that were also within a 10 kb (discrete) window. Pruning was performed using bcftools +*prune* and options were chosen as suggested values within the manual (Li and Durbin 2009). For our admixture analysis, we used Plink v1.9 (Purcell et al. 2007). The optimal number of clusters across the retained 303,511 unlinked nuclear SNPs was determined by the lowest cross validation error. Across the full spectrum of all retained variant and invariant sites, we then used PIXY v1.2.7 (Korunes and Samuk 2021) to estimate genetic differentiation (*F*_ST_) and nucleotide diversity (π) within 100 kb nonoverlapping windows for each population.

### 2.4 Demographic History and Evolutionary Relationships

To infer demographic histories and evolutionary relationships across Canadian beluga, we took a multi‐tiered approach. First, we used PSMC (Li and Durbin 2011) to infer changes in the effective population size (*N*_e_) over an evolutionary time extending several million years into the past. Notably, this programme has been demonstrated to be accurate for inferences of demographic events that occurred in the more ancient past (Li and Durbin 2011; as reviewed by Nadachowska‐Brzyska et al. 2022). The PSMC programme applies the sequential Markovian coalescent to blocks of heterozygosity to estimate the rate of coalescence across unliked regions of single genomes (Li and Durbin 2011). For PSMC, we selected the sample from each population with the highest coverage and ran the programme for 100 iterations for each sample. After testing an array of patterns, we chose a time segment for each sample of 4 + 30 × 2 + 4 + 6 + 10 as this pattern provided the most detailed results. We also employed SMC++ (Terhorst et al. 2017) to gain insight regarding fluctuations in *N*_e_ that spanned between approximately two thousand and 100 kya. Like PSMC, SMC++ uses the sequential Markovian Coalescent, but also incorporates information from the SFS (Terhorst et al. 2017), which can be revealing of more recent demographic history (Beichman et al. 2018). We generated the unfolded SFS of each population using easySFS (Gutenkunst et al. 2009) in Python v3.10.2 (Van Rossum and Drake 1995), and ran SMC++ for 10 iterations for each population. Because selection can distort demographic inferences (Ewing and Jensen 2016; Marchi et al. 2021), we also used bcftools *view* and the reference genome annotation file to mask coding regions from demographic inferences with both PSMC and SMC ++. For all demographic analyses, we also used a generation time of 32 years, estimated from the single dentinal growth layer per year (Garde et al. 2015), and a mutation rate of 1.65 × 10^−8^, which was estimated by Westbury et al. (2019). We also used SMC++ *split* to infer the length of time in isolation for the SLE, due to the population's current endangered status.

Last, we used BEAST2 v2.5.1 (Bouckaert et al. 2019) to test hypotheses of the evolutionary relationships across the four Canadian beluga populations. We ran BEAST2 on 303,511 unlinked nuclear SNPs that were used for our Admixture analysis. In BEAST2, we implemented a HKY85 substitution model (Hasegawa et al. 1985), a burn‐in of 10,000 generations, a Yule model prior, and 10 million MCMC iterations. In particular, we used the HKY85 substitution model for its combined simplicity and transition‐transversion bias correction (Hasegawa et al. 1985). The maximum clade credibility tree was then generated using the posterior probabilities for mean heights, and the Pacific bottlenose dolphin was used as the outgroup.

### 2.5 Inbreeding

To know if differences in homozygosity varied across beluga populations, and to assess if homozygosity could be due to recent inbreeding, we used bcftools v1.11 *roh* to estimate runs of homozygosity (ROH) across genomes (779,009 variant sites). This module implements a hidden Markov model and identifies stretches of autozygosity (Narasimhan et al. 2016), which we estimated from genotype likelihoods (‐G30), a derived allele frequeny of 0.4 as suggested by the authors (Li and Durbin 2009), and without the use of a genetic map. To visualize ROH, we plotted the output from bcftools *roh* across individuals for the longest and shortest chromosomes using R package detectRUNS (Biscarini et al. 2019). We then used custom scripts to organize ROH into one of five different size bins for each individual: ROH greater than 5 Mb, ROH between 5 and 1 Mb, ROH between 1 Mb and 500 kb, ROH between 500 kb and 100 kb, and ROH less than 100 kb. We chose these bin sizes as to be comparable to similar studies such as Nigenda‐Morales et al. (2023) and in accord with Ceballos et al. (2018). We performed this step because the lengths of ROH are indicative of the timing and extent of inbreeding and genetic drift. Generally, ROH greater than 5 Mb tend to reflect inbreeding within the last several generations, and shorter bins reflect inbreeding from the more distant past or ROH that have formed through sustained genetic drift (as reviewed by Ceballos et al. 2018). From these data, we estimated the mean length and number of ROH for each individual, as well as the total fraction of genomes found in ROH (FROH) (Tables S2 and S3) using custom scripts in bash and base R (R Core Team 2021) and a total genome size of 2.35 Gb (Jones et al. 2017). We also used vcftools *het* to estimate the inbreeding coefficient (*F*: Hom_observed_ − Hom_expected_/*N*_total_ − Hom_expected_) of all samples together to assess relative levels of inbreeding (Tables S2 and S3). We note that this statistic is aligned with FHOM statistics of inbreeding via comparisons of observed and expected homozygosity (Lavanchy et al. 2024). Last, we estimated the time to the most recent common ancestor (tMRCA) using the longest ROH and mean length ROH of each individual given a generation time of 32 years (Garde et al. 2015) and a recombination rate of 1 cM/Mb as in Thompson (2013) and Crossman, Fontaine, and Frasier (2024), Crossman, Hamilton, et al. (2024).

### 2.6 Mutation Load and Genetic Purging

To estimate mutation load, we used SnpEff (Cingolani et al. 2012), which annotates sites according to the predicted impact of mutations on protein structure. As in Grossen et al. (2020), we categorized predicted impacts as ‘modifier’, ‘low’, ‘moderate’ and ‘high’. Modifier mutations were considered those found in intergenic regions and high impact mutations were those predicted to lead to premature stop codons or frameshift mutations. Low and moderate impact mutations were those predicted as synonymous and missense mutations, respectively. We considered the derived allele at each site to be deleterious because derived alleles are often deleterious in nature (Keightley and Lynch 2003). Although we acknowledge that nonsynonymous changes can segregate under positive selection, SnpEff has served as a popular method of annotating putatively deleterious genetic variation in natural populations (as in Grossen et al. 2020; Humble et al. 2023; Chen et al. 2025; Xiao et al. 2026) and may even be more suitable for smaller sample sizes compared to more conservative approaches such as Protein Variation Effect Analyser (Piñeiro et al. 2025). We also note that other common methods of estimating mutation load, such as the Genome Evolutionary Profiling Rate, also require certain assumptions, such as gene turnover (as reviewed by Bertorelle et al. 2022). To strengthen our SnpEff analyses, we characterized all predictions of mutation impacts using transcript and protein sequence data from the beluga reference assembly.

Following annotation, we estimated the proportion of heterozygous and homozygous genotypes across all sites that segregated each category of predicted mutation impact using vcftools v0.1.16 (Danecek et al. 2011) *hardy*. This was performed for each sample. We also calculated the frequency of deleterious alleles for each sample using custom scripts in bash and R. Genetic purging was assessed using the *R*_*xy*_ statistic for each pair of beluga populations and for each mutation impact category predicted from SnpEff. This statistic is the ratio of the frequency of an allele *i* at a single locus in population_x_ compared to the frequency of that same allele *i* at that same locus in population_y_ (Do et al. 2015). This statistic is also widely used to test for genetic purging (e.g., Grossen et al. 2020; Ochoa and Gibbs 2021; Kardos et al. 2023; Quinn et al. 2024), where the expectation is that smaller populations tend to more effectively purge high impact (lethal) alleles, while accumulating lower impact mutations due to decreased selection efficacy relative to genetic drift. For each comparison involving the SLE, we placed this population first due to its smaller *N*_e_.

### 2.7 Inbreeding Depression in the SLE

Due to the high presence of neonate mortality in the SLE, we sought to test for signals of inbreeding depression. We divided the samples of the SLE into two groups—the nine samples that were biopsied as adults (after removing a sample with strong NVK ancestry) and the eight samples that were necropsied as neonate mortalities. To confirm that these samples were from one population, we performed both relatedness and principal component analyses with R package rCNV (Karunarathne et al. 2023) using the method described by Yang et al. (2010) and Plink, respectively, across 1,796,656 segregating sites. We also estimated the relationships across samples using BEAST2 v2.5.1 with the same settings described previously. To assess inbreeding, we again used both vcftools *het* and bcftools *roh* ‐G30 with a derived allele frequency of 0.4. In addition to inbreeding statistics, we estimated mutation load as described above. For example, we once again estimated the frequency of the deleterious alleles, as well as the proportion of sites that were either heterozygous or homozygous for the deleterious allele for each sample. The rationale behind these analyses was that inbreeding depression in the SLE should be reflected by more inbred neonate mortalities with higher mutation loads.

### 2.8 Identifying Loci Potentially Linked to Neonate Mortality in the SLE

In theory, causal variants linked to neonate mortality in the SLE might be expected to be fixed derived (assumed deleterious) across neonates and fixed ancestral in the adults. However, different haplotypes comprised of multiple loci with epistatic interactions could also contribute to neonate mortality (as in Robinson et al. 2019; Stoffel et al. 2024). We analysed all coding sites that were absent of deleterious homozygotes in adults but had at least one deleterious homozygote in the neonate mortality samples. These sites were identified using custom scripts in bash and R.

To explore the potential of epistatic interactions that correlated with neonate mortality across these sites, we used the R package WISH‐R (Carmelo et al. 2018). This programme implements the Weighted Interaction Snp Hub (WISH) approach (Kogelman and Kadarmideen 2014) to identify clusters of alleles that covary and are associated with a given phenotype after accounting for linkage disequilibrium (LD) (Carmelo et al. 2018). We tested epistatic models across all loci using a Generalized Linear Model with a binomial link in WISH‐R, setting case (neonate mortality) and control (biopsied adult) phenotypes as the response variable. Estimates of epistasis across loci were then rescaled to correlations and the resulting dissimilarly matrix was used to generate gene dendrograms by WISH‐R. As recommended by the programme tutorial (https://github.com/QSG‐group/wish), we trimmed branches of the dendrogram that had a similarity above 0.6. This reduced the original output of 10 unique gene modules to eight, each of which strongly aligned with a different neonate mortality sample (Figure 4c). Input genotype data were generated using Plink, and we filtered data based on LD values greater than 0.75.

Once we identified gene modules, we performed independent GO enrichment analyses for each module using R package WebGestalt R (Liao et al. 2019). We performed this step to assess if the loci within each gene module were associated with any common functions. To test for terms that could be nonrandomly associated with the epistatic loci of gene modules, we used an over‐representation approach of GO terms, selecting the top 10 results based on the highest enrichment ratios—the ratios of the observed counts to the expected counts given a particular gene set. For GO enrichment, we used gene names that were attained by intersecting the genomic coordinates of each locus of each gene model with exons in the beluga reference assembly using bedtools *intersect*. We used the cow ( *Bos taurus* ) genome as a background gene set in WebGestalt R and we retained all redundant terms. Significant GO enrichment was considered at a False Discovery Rate of less than 0.05 after Bonferroni correction. The top term for each module is shown in Figure 4d, and all results from GO enrichment analyses are shown in the Supporting Information. Note that details of specific commands used for all analyses in our methods can be found at https://github.com/richardorton/CanadianBeluga_DemographicHistory_MutationLoad.

### 2.9 Interpreting Variation in Our Datasets

To compare and contrast variation across Canadian belugas and within the SLE, we implemented Bayesian models in the R package, BANOVA (Dong and Wedel 2017). Specifically, we tested the effect of population and group assignment on estimates of genetic diversity, inbreeding and mutation load in a framework analogous to hierarchical ANOVA. Our models were coded in the STAN language and were fit by running three chains in parallel, each with 10,000 MCMC iterations. For each model, we used a warmup of 1000 iterations and checked model performance and convergence by visualizing trace plots and by making sure that models passed both Geweke's and Heidelberg and Welch convergence diagnostics in BANOVA. Priors were assumed by BANOVA and we *z*‐standardized all of our response variables using custom scripts in R.

To quantify variation in genetic diversity across populations, we ran six independent models with six respective response variables: (1) *F*_ST_, (2) π, (3) ROH, (4) *R*_*xy*_, (5) heterozygosity at sites segregating deleterious variation and (6) homozygosity at sites segregating deleterious variation. Population was set as the predictor variable across all models, and we additionally implemented mutation impact category in a hierarchical design for the last four models to quantify the effect of mutation impact and its interaction with population on genetic diversity. For instance, the interaction effect of mutation impact category with population on *R*_*xy*_ could indicate genetic purging, where a deficit of high impact deleterious mutations along with surpluses of lower impact mutations in the bottlenecked population indicates genetic purging (Do et al. 2015). For our analyses within the SLE, we used models to test the effect of group (neonate mortality or adult) on FHOM and ROH greater than 2.5 Mb. We also reran models five and six with group as the predictor variable. For each model, we estimated the effect size of the predictor variable, along with the 95% credible interval. The BANOVA tutorial proposes there to be a *significant* effect of the predictor variable if the 95% credible interval does not intercept with zero (Dong and Wedel 2017). Our results were visualized using ggolot2 in R (Wickham 2016). The overall effect of each predictor variable, as well as the mean and 95% credible interval of the posterior probability of each level of predictor variable are shown accordingly in the Supporting Information.

## 3 Results

### 3.1 Genetic Differentiation

We uncovered monophyly (Figure 1b and Figure S1a) and four genetic clusters that partitioned according to geographic sampling locations (Figure S1b). We note that a single individual sampled in the Saint Lawrence Estuary showed strong ancestry of NVK (Figure S1b). However, this individual was later determined to have unexpectedly died from trauma, introducing additional uncertainty as to its origin. We thus removed it from all downstream analyses. Even with this individual, however, the SLE was supported as sister to all other populations (Figure 1b and Figure S1a) and was the first cluster to differentiate from the others at *K* = 2 (Figure S1b). The SLE also showed the highest pairwise estimates of genetic differentiation, with *F*_ST_ values ranging from 0.10 to 0.12 (Figure S2).

### 3.2 Demographic History

To assess the extent of isolation and the historical population sizes for each of the four Eastern Canadian beluga sampled here, we inferred changes in *N*_e_ that occurred over the last three million years, a time frame that approaches the presumed split of beluga and narwhal ( *Monodon monoceros* ) (Westbury et al. 2019). We used PSMC (Li and Durbin 2011) and SMC++ (Terhorst et al. 2017) to infer *N*_e_ across overlapping historical and more contemporary time frames, respectively (as reviewed by Nadachowska‐Brzyska et al. 2022). For each population, we found a reduction in *N*_e_ that began at the onset of the Last Glacial Period (LGP) during the Pleistocene approximately 125 thousand years ago (kya) (Figure 1c and Figure S3). For example, the *N*_e_ of NVK and BIS populations peaked 103,674 and 125,910ya at values of 12,378.7 and 11,609.1, respectively. Peak *N*_e_ estimates for the EHA and SLE populations also occurred at 116,987 and 114,528 ya at values of 12,091.4 10,556.3, respectively. It was during this period where the evolutionary trajectory of each population became idiosyncratic. Splitting analyses via SMC++ indicated that the SLE, in particular, became isolated from other Eastern Canadian beluga between 48 and 50 kya during the middle of the LGP (Figure S3).

### 3.3 Genetic Diversity

Among the four Eastern Canadian beluga assessed here, estimates of π were comparatively low for the SLE. Moreover, π was quite similar for the other three lineages (Figure S4), potentially reflecting closely shared evolutionary histories or similar contemporary estimates of *N*_e_ (Müller et al. 2026). For example, the mean value of π across all windows for the SLE was 0.0017 while the mean values of π across all windows for NVK, BIS and EHA were 0.0022, 0.0022 and 0.0021, respectively. Individuals from the SLE also had genomes composed of a higher number of fixed sites (Figure S5). Moreover, individuals from the SLE had genomes comprised of ROH (Figures S6 and S7) that were more frequent (Figure S8) and longer (Figure S9) than individuals sampled from other locations. Overall, individuals in the SLE have a greater fraction of the genome captured in ROH compared to those sampled from other lineages (Figure 1d). In particular, the greater number of ROH over 1 Mb and up to 9 Mb suggests both increased genetic drift and increased inbreeding in the SLE that has occurred throughout the history of SLE up to the last few generations (Figure S2). This is reflected in our estimates of the tMRCA where the longest ROH of most individuals coalesces between 200 and 400 ya for the SLE and between 500 and 1000 ya for other Canadian belugas (Table S2). Meanwhile, the mean length ROH coalesces on average approximately 4000 ya for the SLE and approximately 6000 ya for the other populations.

### 3.4 Mutation Load and Genetic Purging

**FIGURE 2:**
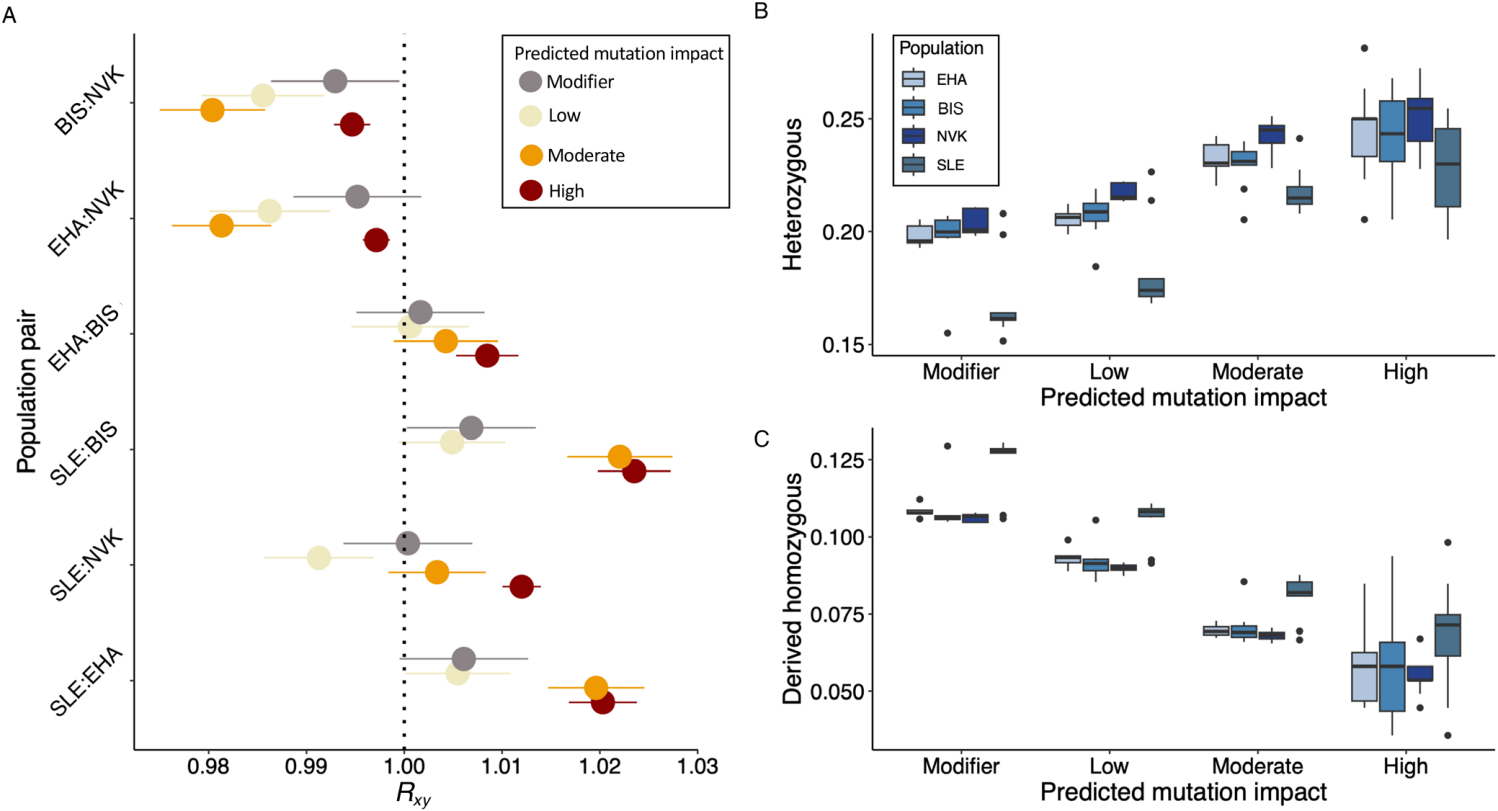
The characteristics of mutation load across Eastern Canadian beluga populations. (A) Dot plots show the estimates of *R*_*xy*_ for each population pair for each predicted mutation impact category. A value of 1, shown as the vertical dashed line, indicates that the frequency of deleterious alleles within individual genomes is similar for each population. Population pair is shown on the *Y* axis, *R*_*xy*_ is shown on the *X* axis, and the predicted mutation impact category is coloured as denoted in the legend. (B) Box plots depicting the proportion of sites across each individual's genome that harbours a deleterious allele in a heterozygous genotype for each predicted mutation impact category. (C) Box plots depicting the proportion of sites across each individual's genome that harbours two deleterious alleles in a homozygous genotype for each predicted mutation impact category. For (B, C), the proportion of sites is shown on the *Y* axis, predicted mutation impact category is shown on the *X* axis, and population is denoted by the colour shown in the legend of (B). Error bars for each plot designate (±) one standard error.

We found that the SLE has accumulated a higher frequency of deleterious mutations compared to other Eastern Canadian beluga (Figure 2a and Figure S10). Although NVK individuals do have a higher proportion of sites segregating deleterious alleles as heterozygotes, which is linked to the potential mutation load (Figure 2b and Figure S11), the SLE has a higher proportion of sites segregating deleterious alleles as homozygotes (Figure S12), leading to the higher overall frequency of deleterious alleles. Using the *R*_*xy*_ statistic, which summarizes the ratios of alleles at homologous loci between two populations, we also found no footprint of genetic purging for any Eastern Canadian beluga given values of *R*_*xy*_ (Figure 2a and Figure S10). In particular, the interaction between mutation impact category and population pair had little effect on overall estimates of *R*_*xy*_, suggesting that selection has not differentially acted upon high impact alleles in any single population. In comparison to other populations, for example, the SLE had a surplus of deleterious alleles for all categories.

### 3.5 Inbreeding Depression

**FIGURE 3:**
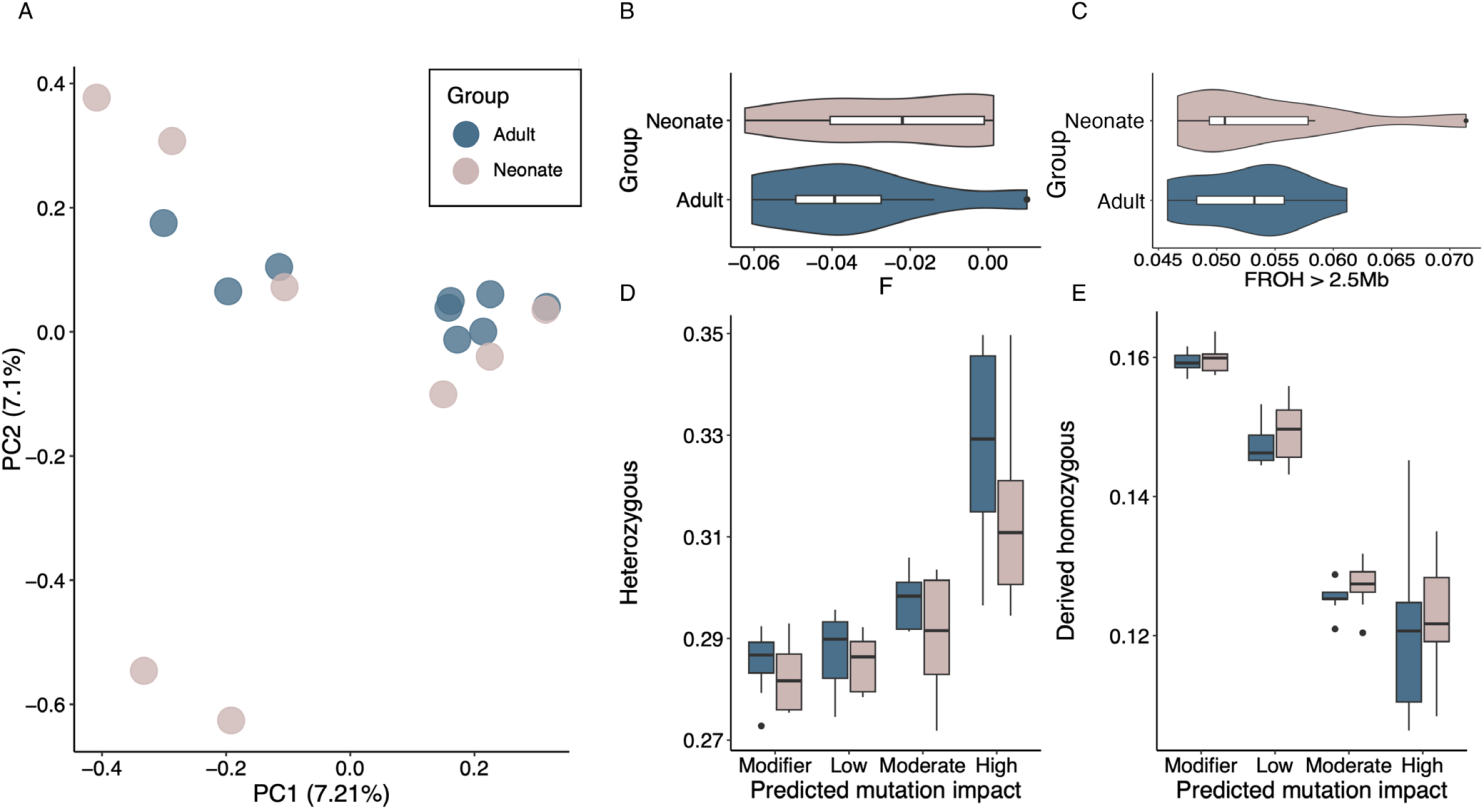
Estimates of inbreeding and mutation load in the SLE. (A) Principal component analysis from segregating sites in the SLE. (B) Violin plots depicting estimates of inbreeding by FHOM for neonate mortalities and biopsied adults. (C) Violin plots depicting estimates of inbreeding by the fraction of genomes captured in runs of homozygosity greater than 2.5 Mb (FROH > 2.5 Mb) for neonate mortalities and biopsied adults. (D) Box plots depicting the proportion of sites across each individual's genome harbouring a deleterious allele in a heterozygote genotype for each predicted mutation impact category. (E) Box plots depicting the proportion of sites across each individual's genome harbouring two deleterious alleles in a homozygote genotype for each predicted mutation impact category. For (C, D), proportion of sites is shown on the *Y* axis, predicted mutation impact category is shown on the *X* axis, and sampling group is denoted by the colour shown in the legend of (A). Error bars for each plot designate the (±) one standard error.

**FIGURE 4:**
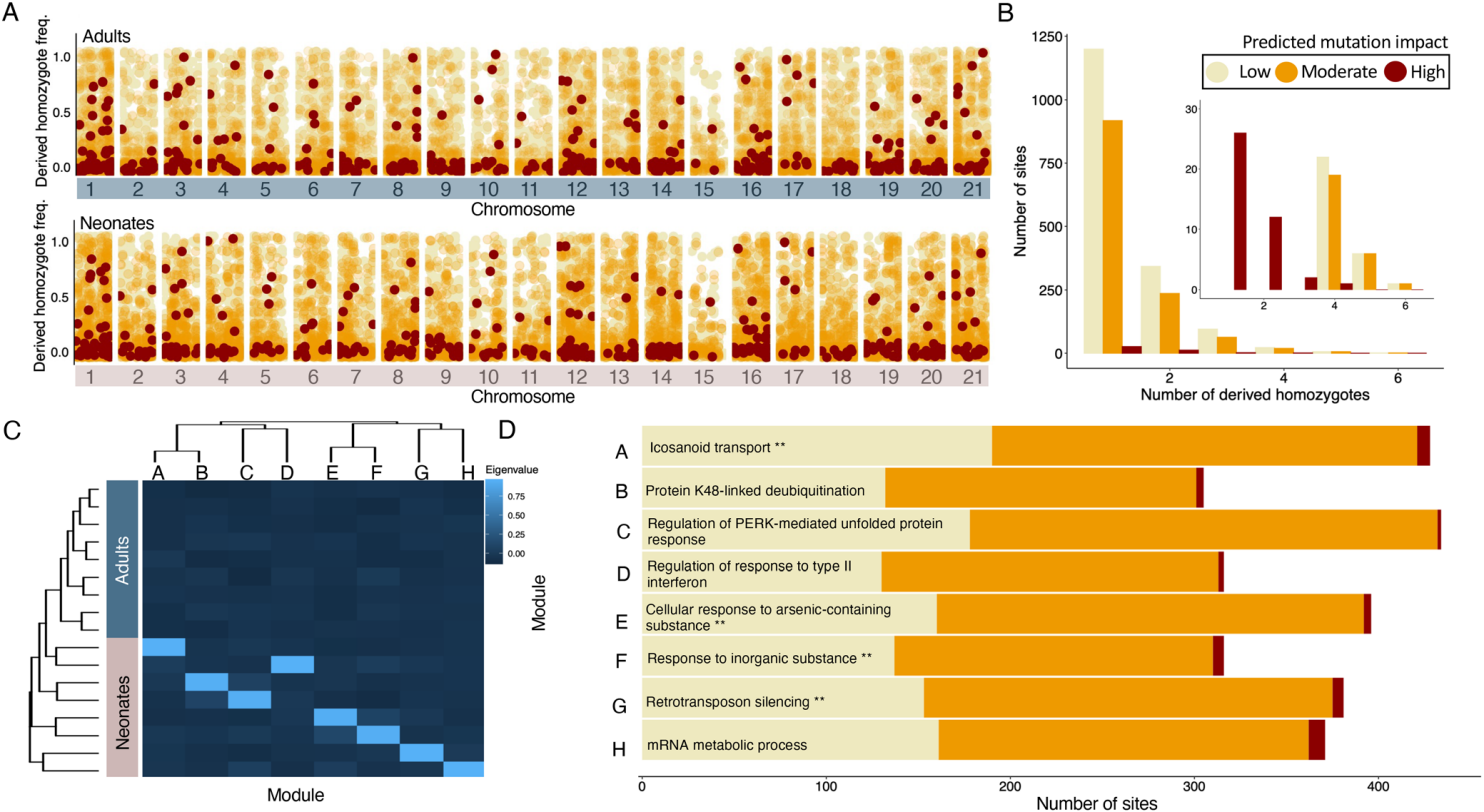
Epistatic interactions across loci homozygous for deleterious alleles in neonate mortalities. (A) The genetic landscapes of the frequencies of derived homozygotes in biopsied adults (top) and in neonate mortalities (bottom). (B) A histogram of the 2497 sites only segregating derived homozygotes in neonate mortalities, depicting the distribution of sites segregating differing numbers of derived homozygotes. Colour denotes the predicted mutation impact category for each homozygote harboured across neonate mortality genomes as shown in the legend. The inset highlights the distribution of sites for rare, derived homozygotes. (C) A heatmap of the epistatic gene modules associated with neonate mortality. Individuals and their genetic relationships are shown on the *Y* axis and the gene modules and their associated dendrogram are show on the *X* axis above. Blue shades indicate the strength of the correlation between gene module and phenotype as depicted in the legend. (D) The top Gene Ontology term for each gene module and associated dendrogram from (C). The number of loci forming each gene module is shown on the *X* axis, with the number per predicted mutation impact category coloured consistently with (A) and (B). Asterisks denote modules with significant associations to cellular responses to inorganic substances. The top 10 Gene Ontology associations for each gene module are shown in the Supporting Information.

To evaluate if neonate mortality, a chief consequence of low genetic diversity and inbreeding depression (Frankham 1995), could be linked to deleterious genetic variation in the SLE, we compared the genomes of eight neonate mortalities and nine biopsied adults sampled from the Saint Lawrence Estuary (Figure 3a and Figure S13a,b). We found no evidence that neonate mortalities were more inbred than adults given estimates of FHOM or the distribution of ROH > 2.5 Mb (Figure 3b,c and Figure S14a,b). However, we did find that neonate mortalities were less heterozygous (and more homozygous) across sites segregating deleterious alleles (Figure 3d,e and Figures S15a,b and S16). Specifically, 2497 sites did not segregate deleterious homozygotes in adults while harbouring at least one deleterious homozygote in neonate mortalities (Figure 4a,b). In many instances, multiple homozygotes were found at these sites across neonate mortalities.

In the SLE, the 2497 sites that differentially segregate deleterious homozygotes clustered into eight unique gene modules (Figure S17) that were comprised of physically unlinked (Figure S18a,b) but also putatively epistatic alleles found across chromosomes (Figure S19). These eight unique gene modules were strongly correlated with neonate mortality (Figure 4c), and half were linked to cellular responses to inorganic substances provided the results of our GO enrichment analyses (Figure 4d and Figures S20–S23).

## 4 Discussion

### 4.1 Genetic Differentiation

The patterns of genetic differentiation and diversity found here broadly reflect independently‐evolving lineages of beluga whale in Eastern Canada, supporting the basis for our comparative sampling design (Figure 1a). In light of two recent genomic studies that resolved clear population structure across a wide geographic sampling of Eastern Canadian beluga (Montana et al. 2024; Müller et al. 2026), we were also able to reference our samples to previously defined populations. For example, our SLE samples belong to the Saint Lawrence Estuary population while EHA samples come from the Resolute Bay/Eastern High Arctic population (Montana et al. 2024). Although referencing samples from BIS and NVK was slightly more ambiguous due to beluga whale migration patterns, those samples most likely trace to the Little and Great Whale Rivers and Hudson Bay Strait Complex populations, respectively (Montana et al. 2024; Müller et al. 2026). For simplicity, we refer to the sampling locations of our study as *populations*, given that they appear to represent previously defined populations with little admixture (Figure S1).

Among our samples, we found increased genetic differentiation for the SLE. This increased differentiation not only mirrors early genetic analyses of Canadian beluga that assessed a major histocompatibility locus (Murray et al. 1999), a mitochondrial d‐loop sequence, and 15 microsatellite markers (de March and Postma 2003); it also aligns with spatial inferences (Bailleul et al. 2012), traditional knowledge of the Inuit (Breton‐Honeyman et al. 2016), morphometric studies (DFO 2023) and isotope analyses (Rioux et al. 2012). Collectively, these studies provide strong evidence that the SLE is more differentiated (or isolated) than other Canadian beluga. This insight could have important conservation implications because an extended period of isolation can lead to an extinction vortex in smaller populations where mutation load and inbreeding cause fitness declines through a feedback loop (Souleet al. 1986).

### 4.2 Demographic History

Estimates of *N*_e_ were strikingly similar for all populations prior to the LGP, suggesting that reductions in *N*_e_ could be artifacts of the stratification of a larger ancestral population. This inference adds to a recent study that hypothesized that differentiation and isolation of beluga populations spanning the Arctic was shaped by sea ice extent during the LGP (Skovrind et al. 2021). Our results are also comparable to populations of Fin whales ( *Balaenoptera physalus* ) that differentiated in the Eastern Pacific during the LGP (Nigenda‐Morales et al. 2023), suggesting broader links between sea ice levels and the evolutionary histories of Arctic and subarctic cetaceans. Our splitting analyses via SMC++ further supports that the SLE became isolated from other Eastern Canadian beluga between 48 and 50 kya during the middle of the LGP (Figure S3). The fossil record shows that belugas were indeed as far south as the Saint Lawrence Estuary by at least the end of the Last Glacial Maximum (LGM) (Harington 2008), and since then, the SLE appears to have maintained a smaller *N*_e_ than other Eastern Canadian beluga (Figure 1c and Figure S3).

### 4.3 Genetic Diversity and Inbreeding

The comparatively small population size and isolation of the SLE, sustained for potentially more than a thousand generations (generation time = 32 years [Garde et al. 2015]), likely accounts for its reduced genetic diversity (Figure S4), greater number of fixed sites (Figure S5), and its high homozygosity. Notably, these are population characteristics representative of both the strong genetic drift and frequent inbreeding common to smaller populations and isolation (Frankham 2018). In particular, the ROH of the SLE range from less than 100 kb to over 8 Mb (Figure 1d). Across generations, long ROH from consanguineous pairings become fragmented into shorter runs by recombination (as reviewed by Ceballos et al. 2018; McQuillan et al. 2008). For instance, ROH between 5 and 1 Mb tend to represent remnants of ROH that have undergone between 10 and 50 generations of recombination (Thompson 2013; Zavarez et al. 2015), while ROH shorter than 1 Mb reflect both ancient inbreeding and genetic drift due to long‐term small population size and isolation (as reviewed by Ceballos et al. 2018).

Although the abundance of ROH between 100 kb and 1 Mb in the SLE highlights the influence of genetic drift through sustained isolation (as in Mooney et al. 2023), the longest ROH of each individual in the SLE (up to 9 Mb) roughly coalesce between six and 13 generations in the past (Table S2). This corresponds to between 200 and 400 ya, a period when hunting pressures would have been high in the SLE (Béland 1996). For comparison, the longest ROH of the other Canadian beluga coalesce between 500 and 1000 ya following the Medieval Warming Period (Goosse et al. 2006). Still, the average ROH in the SLE is estimated to have formed approximately 4000 ya, suggesting that inbreeding was more common in the SLE in the distant past. It may therefore be most informative to consider genetic diversity and homozygosity in the endangered SLE from the perspective of both ongoing genetic drift and inbreeding that has occurred across the history of the SLE due to sustained isolation.

### 4.4 Mutation Load and Genetic Purging

The extent of ROH in SLE individuals, in particular, has implications for the conservation of Eastern Canadian beluga because the fitness consequences of deleterious mutations and homozygosity are linked. Deleterious mutations are often at least partially recessive and tend to require homozygosity for their expression (Yang et al. 2017). Populations with high homozygosity, like the SLE, could thus be susceptible to genetically based declines via the expression of deleterious alleles. Conversely, populations with shorter and fewer ROH, such as NVK (Figures S8 and S9), can shelter high frequencies of recessive deleterious alleles across swathes of heterozygous loci without immediate fitness costs.

Despite any obvious genomic consequences, such as high rates of neonate mortality (DFO 2023), estimates of the potential mutation load in the NVK does deserve attention because future population contractions could rapidly convert the high potential mutation load to a high realized mutation load (Kyriazis et al. 2021). Meanwhile, the high proportion of sites homozygous for deleterious alleles, an approximation of the realized mutation load in the SLE (Figure 2c and Figure S12) could account, at least in part, for the population's current conservation status. In addition to the abundance of short ROH, the increased homozygosity found across mutations of all predicted impacts on protein structure, including mutations segregating across intergenic loci (Figure 2c and Figure S12), implicates the impact of genetic drift. Feasibly, the long‐term relatively small *N*_e_ in isolation allowed genetic drift to outpace purifying selection in the SLE (Kimura et al. 1963), even across genomic targets with putatively high selection coefficients (e.g., high impact mutations).

The consensus across current genomic studies is that genetic purging in target populations reduces the frequency of high impact deleterious alleles while maintaining surpluses of lower impact mutations relative to populations with larger population sizes (Do et al. 2015). This pattern results from the loss of recessive *lethals* that are exposed to selection in bottlenecked populations despite the diminished efficacy of selection that simultaneously allows for lower impact deleterious alleles to accumulate (Kimura et al. 1963). Studies have used *R*_*xy*_ to infer genetic purging in bottlenecked populations of taxa ranging from plants (Wang et al. 2024) to mammals such as Alpine Ibex ( *Capra ibex* ) (Grossen et al. 2020), mountain gorilla ( *Gorilla beringei* ) (Xue et al. 2015) and Orca ( *Orcinus orca* ) (Kardos et al. 2023). One key difference between those studies and ours, however, is that the SLE shows comparatively little genomic evidence of frequent inbreeding within the most recent generations.

Although the probability of effective genetic purging is widely accepted to depend on a population's demographic history (Caballero et al. 2017; Pérez‐Pereira et al. 2022; Xie et al. 2022), genetic purging could be more directly influenced by rates of inbreeding (Hedrick 1994; López‐Cortegano et al. 2021). Whole genome data reveals that deleterious mutations tend to be enriched in long ROH that are formed through very recent inbreeding before recombination fragments those ROH (Szpiech et al. 2013; Zhang et al. 2015). Purifying selection that targets deleterious variation in long ROH could therefore purge clusters of linked deleterious alleles (but see Bersabé et al. 2016). Indeed, evolution at any locus segregating a deleterious allele is affected by selection at linked loci (as reviewed by Charlesworth 2012). In addition to simulations (Szpiech et al. 2013), recent empirical data also illustrate that the signature of genetic purging is most evident within longer ROH anticipated to have formed through recent inbreeding (Orton et al. 2024). Purifying selection that targets unlinked deleterious alleles distributed across shorter ROH formed through genetic drift or more ancient inbreeding might less effectively remove clusters of deleterious alleles, slowing the rate of genetic purging. Because the strength of selection (relative to genetic drift) is inversely proportional to *N*_e_ (Kimura et al. 1963), the probability of effective genetic purging also declines with decreasing *N*_e_ (Pérez‐Pereira et al. 2022). As previously suggested through the use of genomic simulations (Xie et al. 2022), populations that have demographic histories marked by sustained small *N*_e_ without frequent inbreeding in the contemporary, such as belugas occupying the Saint Lawrence Estuary, could be most susceptible to extinction vortex.

### 4.5 Inbreeding Depression and Genetic Drift

In addition to pervasive infectious disease and cancer in the SLE (Larrat et al. 2024), neonate mortality has gained recent attention in light of a rate that has been rising since monitoring began in 1983 (Mosnier et al. 2014). Because neonate mortality is often linked to inbreeding depression, we sought to explore the potential of a genetic basis for neonate mortality in the SLE. Although we did not find neonate mortalities to be more inbred than biopsied adults, we did find 2497 sites that did not segregate as homozygotes in biopsied adults, and thus, inbreeding depression in the SLE could be due to locus‐specific variation, such as in the Isle Royale wolf ([ *Canis lupus* ] Robinson et al. 2019). Notably, the majority of these 2497 sites segregated low or moderate impact mutations (Figure 4b), which can have critical synergistic effects in smaller populations (Robinson et al. 2019; Stoffel et al. 2024). Foundational studies of population genetics posited that fitness losses through the synergetic effects of lower impact mutations are more likely to outpace purifying selection than recessive lethals (Kimura and Maruyama 1966; Ohta 1973). Hedrick (1994) also used theoretical simulations to demonstrate that deleterious variation is more likely to become fixed when the impacts are sublethal, ultimately decreasing the probability of effective genetic purging.

Although we present the latter with some caution due to the noise associated with analyses of epistasis and gene enrichment using smaller sample sizes, we also note the results of this exploratory analysis as relevant because industry operating along the Saint Lawrence Estuary has discharged toxic substances into the local environment for decades (Lean 2000). In particular, polychlorinated biphenyls (PCBs), dioxins and an array of metals such as arsenic are found at high levels in the Saint Lawrence Estuary (Carignan et al. 1994). Not only have these toxins been found in resident belugas at appreciable levels (Allan 1988; Béland et al. 1993), but genes linked to PCB contamination have been recently identified as differentially expressed in the SLE compared to belugas in Alaska (Simond et al. 2025). A study by Vaudreuil et al. (2024) also documented alarming levels of pharmaceutical pollution in the Saint Lawrence Estuary, which validates a fifth gene module in our study that is associated with ‘cellular response to antibiotics’ (Figure S20). Although causes of death for adult belugas in the SLE have been largely attributed to infectious disease and cancers, less has been affirmed over the cause of neonate mortality (Lair et al. 2016). Our study suggests that at least some of the SLE neonate mortalities could be due to the interaction of deleterious homozygous genotypes and toxins in the environment. The increasing rate of neonate mortalities in the SLE (Béland et al. 1993; Lair et al. 2016) could also be due to increases in the frequencies of deleterious alleles caused by ongoing genetic drift and reduced selection, facets of population isolation (Frankham 2018; Pinto et al. 2024).

## 5 Conclusion

Studies seeking to understand the factors underlying genetic purging have generally concluded that the process hinges on the rate of population contraction and the size of the ancestral population (Caballero et al. 2017; López‐Cortegano et al. 2021). Because no beluga population sampled in our study shows a signal of genetic purging, and because the populations are only recentl*y* differentiated, we are unable to directly address this supposition here. However, the lack of frequent inbreeding within the last several generations coupled with the absence of genetic purging in the SLE does hint that inbreeding could directly impact the efficacy of genetic purging in natural populations. Our study supports simulations showing that the probability of effective genetic purging is ‘highly dependent upon the genetic basis of inbreeding depression’, (Hedrick 1994), and it complements empirical studies of genetic purging that occurred through inbreeding (Grossen et al. 2020; Orton et al. 2024) in that our results characterize an absence of genetic purging without strong ongoing inbreeding in a natural population. In turn, our study adds context as to how demographic history can impact genetic purging while shaping conservation risk.

Broadly, the gradual and sustained reduction in *N*_e_ of the SLE appears to underpin the accumulation of deleterious genetic variation, indicating a population size that is likely problematic. Still, we note here that estimates of *N*_e_ in the SLE (Müller et al. 2026) are an order of magnitude higher than those of the North Atlantic right whale (Crossman, Hamilton, et al. 2024) and the Southern resident killer whale (Kardos et al. 2023), for which rates of inbreeding within the last several generations are strikingly high. In the other populations of beluga, we also found increases in *N*_e_ that followed deglaciation during the LGM (Figure S3). If this signal is not due to population expansion, the increases in *N*_e_ are most likely due to secondary contact between previously isolated beluga that coincides with sea ice extent. Recent population genomics analyses have inferred gene flow across some Eastern Canadian beluga populations (Montana et al. 2024; Müller et al. 2026), suggesting the latter. Because the demographic histories of multiple populations of Arctic and subarctic cetaceans, including narwhal (Westbury et al. 2019; de Greef et al. 2024), Bowhead ( *Balaena mysticetus* ) (Phillips et al. 2013; Cerca et al. 2022; de Greef et al. 2024), Fin (Nigenda‐Morales et al. 2023) and beluga whales (Westbury et al. 2019; Skovrind et al. 2021; Müller et al. 2026), have been shaped by sea ice extent, it may be pertinent to monitor the population dynamics of beluga in Eastern Canada as sea ice extent is reduced via climate change. Any gene flow into the SLE, for instance, could further reduce the efficacy of genetic purging via the introduction of additional deleterious genetic variation (Kyriazis et al. 2021). Our data could be used to establish a baseline for the genetic monitoring of beluga in Eastern Canada, which could help inform conservation decisions and strengthen our understanding of the dynamics of isolated populations in nature.

## Author Contributions

This manuscript was conceived and designed by R.W.O. and T.R.F., S.H.F., R.M., C.J.G. and T.R.F., all contributed samples that were used for data analyses. R.W.O. performed the data analyses, and R.W.O., T.R.F., C.J.G., C.M. and S.H.F. all contributed towards writing the final version of the manuscript.

## Funding

Data collection and funding for the sequencing and analyses of this project were provided through Genome Atlantic and the Department of Fisheries and Oceans Canada.

## Ethics Statement

All protocols and procedures employed were ethically reviewed and approved by the Department of Fisheries and Oceans Canada.

## Conflicts of Interest

The authors declare no conflicts of interest.

## Supporting information


**Figure S1:** (A) Cladogram including all individuals of the four beluga populations generated with BEAST2. The Indo‐Pacific bottlenose dolphin was used as the outgroup and the sampling site (Population) is coloured as denoted in the legend. (B) Ancestry coefficients given by Admixture for values of *K* = 2 to *K* = 4. Sampling site is labelled along the *X* axes and genetic clusters are denoted with the different shades of blue aligned with the legend in (A).
**Figure S2:** Violin plots of genome‐wide estimates of *F*_ST_ in 100 kb nonoverlapping windows (*Y* axis) for each population pair (*X* axis). Median estimate are shown for each population pair at the top. Results from Bayesian analyses for the effect of population on estimates of *F*_ST_ shown to the right. The posterior mean is shown as the dot and whiskers indicate the 95% credible interval. The posterior mean and upper and lower credible interval bounds are shown in the table for the specific effect of each population.
**Figure S3:** The changes in *N*_e_ since the Last Pleistocene Glaciation inferred with 10 iterations of SMC++. We used a generation time of 32 years and a mutation rate of 1.65 × 10^−8^. The dark grey shaded area denotes approximately the Last Glacial Period, and the lighter shaded area shows approximately the Last Glacial Maximum. Vertical lines show the estimated times of divergence between the SLE and the other populations, coloured according to the legend.
**Figure S4:** Split violin and box plots depict genome‐wide estimates of nucleotide diversity (π) in nonoverlapping 100 kb windows (*Y* axis) for each population (*X* axis). Results from Bayesian analyses for the effect of population on estimates of nucleotide diversity. The posterior mean is shown as the dot and whiskers indicate the 95% credible interval. The posterior mean and upper and lower credible interval bounds for the effect of each population are shown in table.
**Figure S5:** The unfolded site frequency spectrum for each population. Populations are coloured as denoted in the legend.
**Figure S6:** Runs of homozygosity plotted across all individuals for chromosome one.
**Figure S7:** Runs of homozygosity plotted across all individuals for chromosome 22.
**Figure S8:** Results from Bayesian analyses for the effect of bin, population and the interaction on estimates of the mean length of ROH per individual. The posterior mean is shown as the dot and whiskers indicate the 95% credible interval. The posterior mean and upper and lower credible interval bounds are shown in tables for each specific effect and interaction.
**Figure S9:** Results from Bayesian analyses for the effect of bin, population and the interaction on estimates of the mean number of ROH per individual. The posterior mean is shown as the dot and whiskers indicate the 95% credible interval. The posterior mean and upper and lower credible interval bounds are shown in tables for each specific effect and interaction.
**Figure S10:** Results from Bayesian analyses for the effect of population pair, mutation impact category, and the interaction on estimates of *R*_*xy*_. The posterior mean is shown as the dot and whiskers indicate the 95% credible interval. The posterior mean and upper and lower credible interval bounds are shown in tables for each specific effect and interaction.
**Figure S11:** Results from Bayesian analyses for the effect of population, mutation impact category and the interaction on estimates of the proportion of heterozygous sites harbouring a deleterious allele. The posterior mean is shown as the dot and whiskers indicate the 95% credible interval. The posterior mean and upper and lower credible interval bounds are shown in tables for each specific effect and interaction.
**Figure S12:** Results from Bayesian analyses for the effect of population, mutation impact category and the interaction on estimates of the proportion of homozygous sites harbouring two deleterious alleles. The posterior mean is shown as the dot and whiskers indicate the 95% credible interval. The posterior mean and upper and lower credible interval bounds are shown in tables for each specific effect and interaction.
**Figure S13:** (A) Estimates of relatedness across all individuals sampled from the SLE including neonate mortalities and biopsied adults. Group is denoted by colour along the *X* axis and relatedness is coloured according to the legend. (B) The percent of genetic variation in the SLE explained by each Principal Component, respectively shown in Figure 3 in the manuscript.
**Figure S14:** Results from Bayesian analyses for the effect of group on (A) FHOM and (B) FROH greater than 2.5 Mb. The posterior mean is shown as the dot and whiskers indicate the 95% credible interval.
**Figure S15:** Results from Bayesian analyses for the effect of predicted mutation impact category, group and the interaction on estimates of the proportion of (A) heterozygous sites harbouring a deleterious allele and (B) the proportion of homozygous sites harbouring two deleterious alleles. The posterior mean is shown as the dot and whiskers indicate the 95% credible interval. The posterior mean and upper and lower credible interval bounds are shown in tables for each specific effect and interaction.
**Figure S16:** The distribution of ROH within each of five size bins for biopsied adults and neonate mortalities in the SLE. Note that results for the biopsied adults slightly vary from those shown in Figure 1 in the manuscript—estimates were taken from different VCF files as described in the methods.
**Figure S17:** (A) The full dendrogram and clustering of epistatic gene modules linked to neonate mortality in the SLE. Note that we show both the original clustering results (top) and the reduced clustering (bottom) after accounting for strongly related clusters (B).
**Figure S18:** (A) The distribution of gene modules across all 22 autosomes of the beluga whale and (B) the pairwise chromosome interactions with the strength and direction of the relationship shown in the legend on the right.
**Figure S19:** Pseudo Manhattan plot of the sum of effect sizes (*Y* axis) for each variant (*X* axis) found to only segregate as a homozygote in the neonate mortalities.
**Figure S20:** The top 10 biological processes for Gene Ontology term enrichment according to the enrichment ratio. Any biological processes with significant *p*‐values after Bonferroni correction are designated with an asterisk.
**Figure S21:** The top 10 biological processes for Gene Ontology term enrichment according to the enrichment ratio. Any biological processes with significant *p*‐values after Bonferroni correction are designated with an asterisk.
**Figure S22:** The top 10 biological processes for Gene Ontology term enrichment according to the enrichment ratio. Any biological processes with significant *p*‐values after Bonferroni correction are designated with an asterisk.
**Figure S23:** The top 10 biological processes for Gene Ontology term enrichment according to the enrichment ratio. Any biological processes with significant *p*‐values after Bonferroni correction are designated with an asterisk.
**Table S1:** Table of samples and their sampling sites, with the assigned group via Admixture or neonate mortality status, sampling year, sex and unfiltered genome coverage.
**Table S2:** Table of homozygosity statistics for each sample with the total sum of all runs of homozygosity (ROH), the total number of ROH, the mean length of ROH, the length of the longest ROH, the fraction of the genome found in all ROH, the inbreeding coefficient given expected and observed homozygosity (FHOM) and the time to the most recent common ancestor (tMRCA) estimated from the longest ROH.
**Table S3:** Table of homozygosity statistics for each SLE sample with the total sum of all runs of homozygosity (ROH), the total number of ROH, the mean length of ROH, the length of the longest ROH, the fraction of the genome found in all ROH, and the inbreeding coefficient given expected and observed homozygosity (FHOM).

## Data Availability

Data have been deposited to NCBI's Sequence Read Archive under accession numbers linked to bioprojects PRJNA1182041 and PRJNA1328577. All scripts used to complete our analyses are available at https://github.com/richardorton/CanadianBeluga_DemographicHistory_MutationLoad.
