# Supplementary material for: Homozygosity Accumulated Through Genetic Drift and Inbreeding Underpins High Mutation Load in an Endangered Beluga Whale Population
Source: Mol Ecol. 2026 Aug 21;35(16):e70521. doi: 10.1111/mec.70521 (PMC13494814; doi:10.1111/mec.70521)
Supplement: Supplementary file 1 [file MEC-35-e70521-s001.pdf]

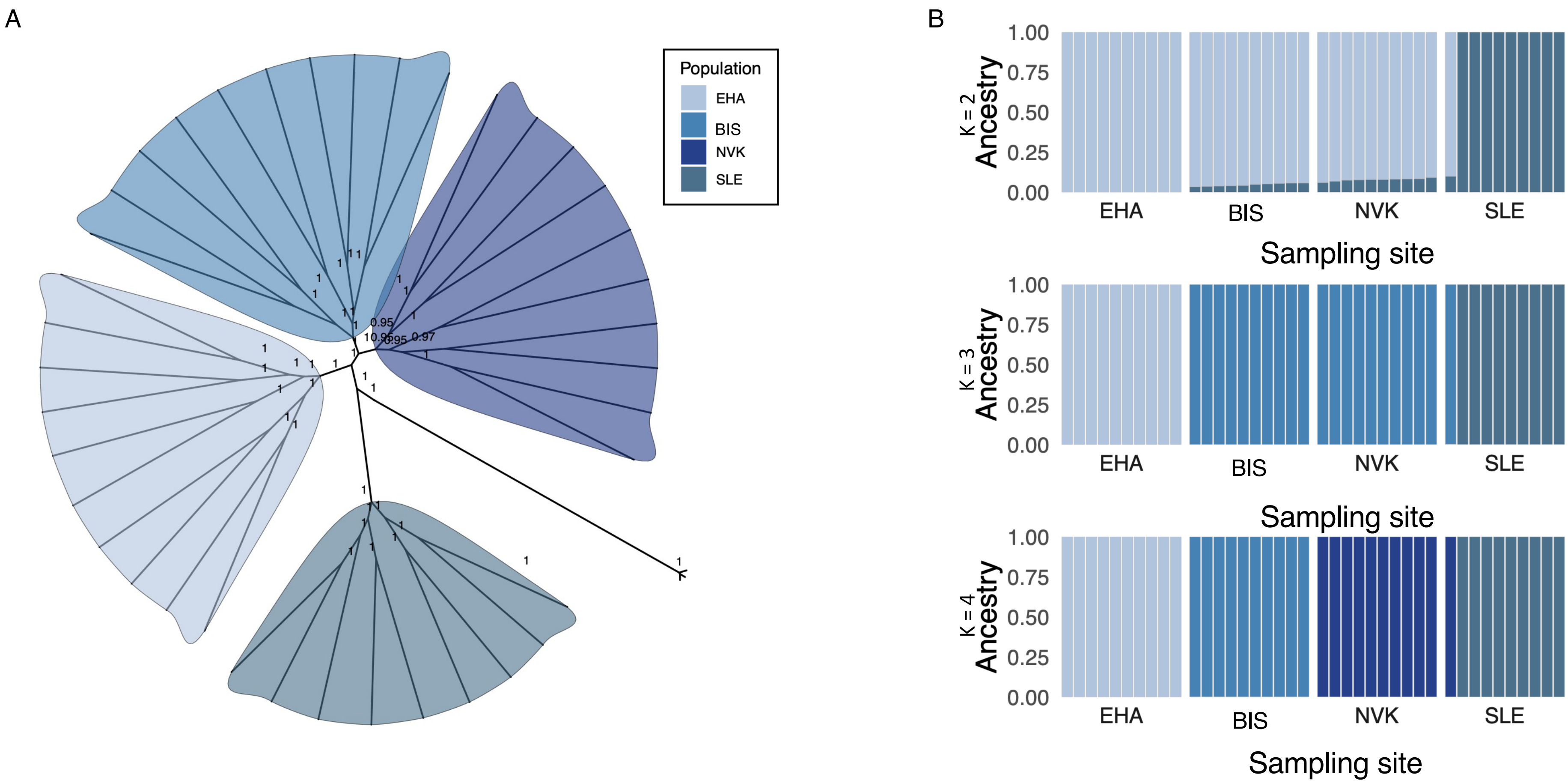
A
Population
EHA
BIS
NVK
SLE
B
K = 2
Ancestry
K = 3
Ancestry
K = 4
Ancestry
1.00
0.75
0.50
0.25
0.00
EHA
BIS
NVK
SLE
Sampling site

Figure S1

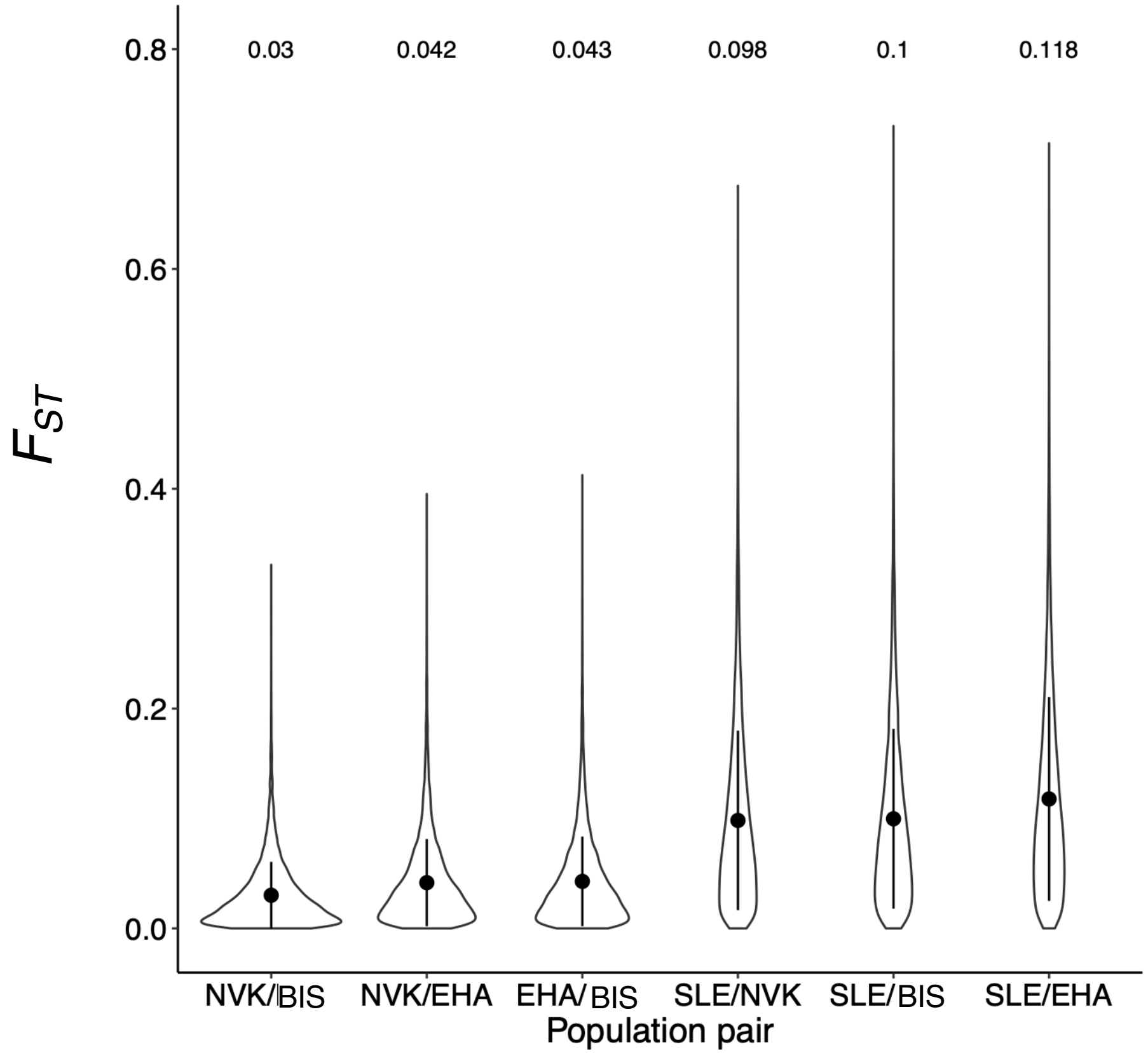

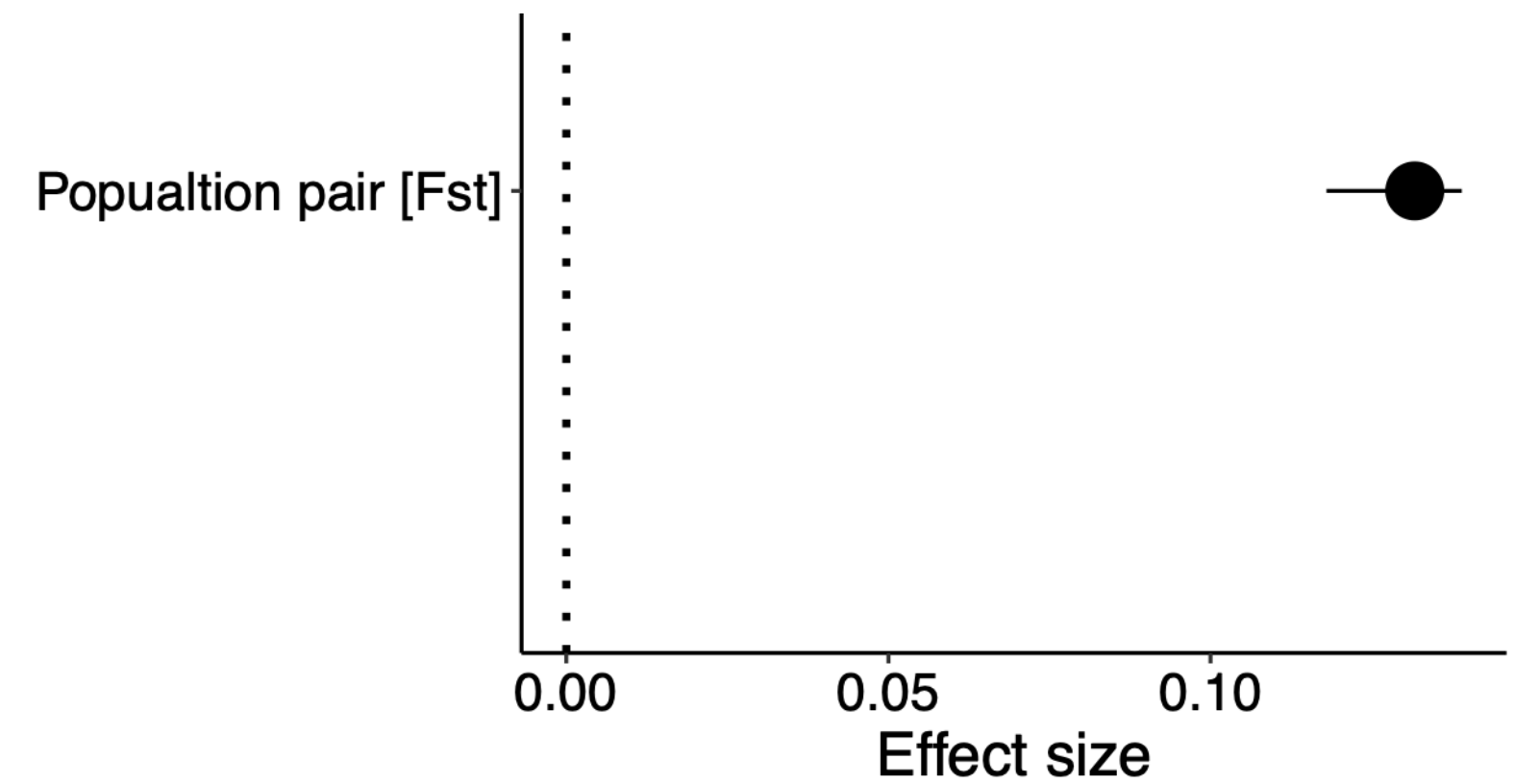

Population pair	mean	2.5%	97.5%
BIS -EHA	-0.50	-0.70	-0.31
NVK-EHA	-0.29	-0.53	-0.06
NVK-BIS	-0.59	-0.87	-0.31
SLE-NVK	0.29	0.11	0.47
SLE-EHA	0.40	0.21	0.58
SLE-BIS	0.17	-0.01	0.35

Figure S2

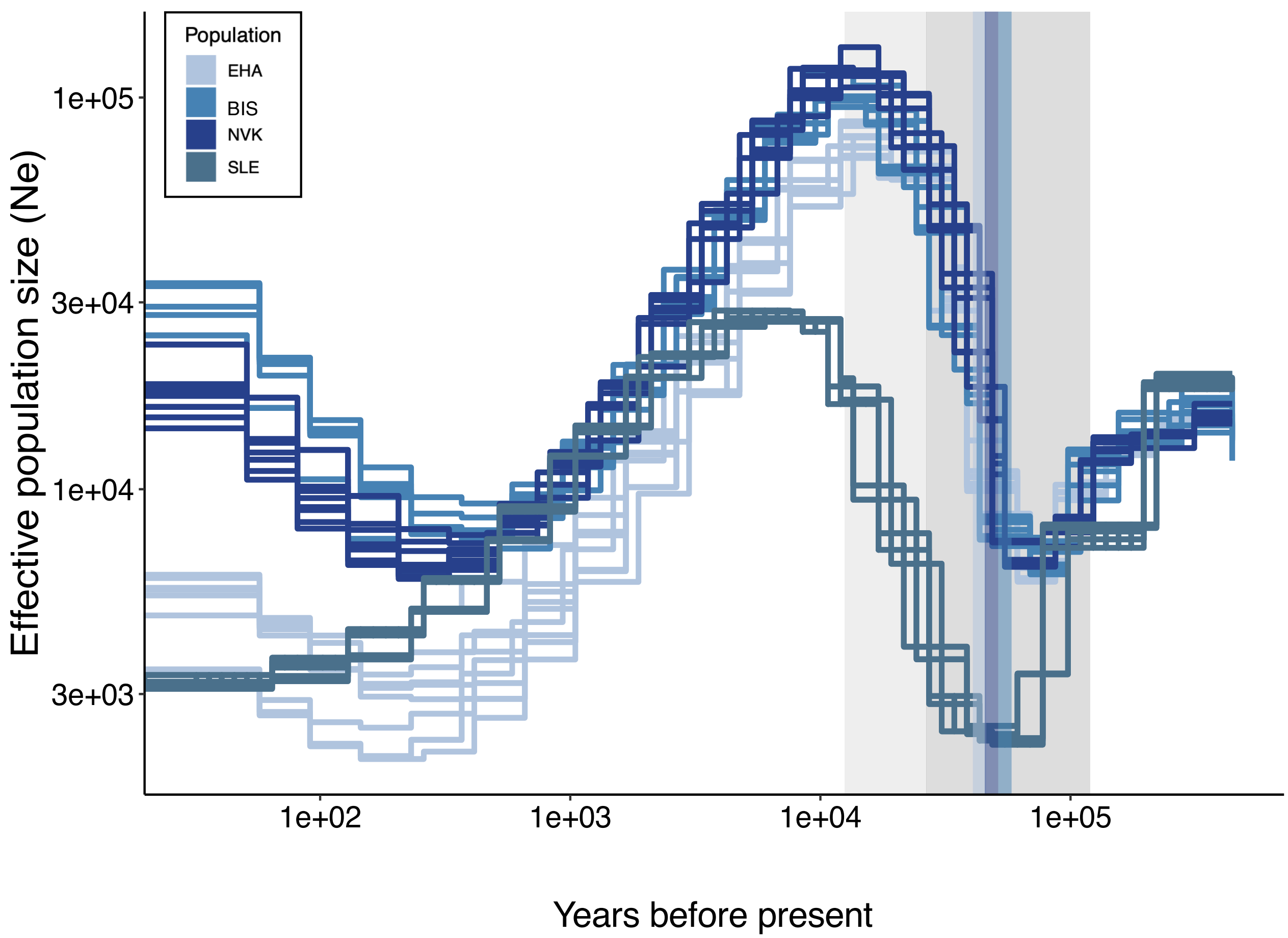
Population
EHA
BIS
NVK
SLE
1e+05
3e+04
1e+04
3e+03
Effective population size (Ne)
1e+02
1e+03
1e+04
1e+05
Years before present

Figure S3

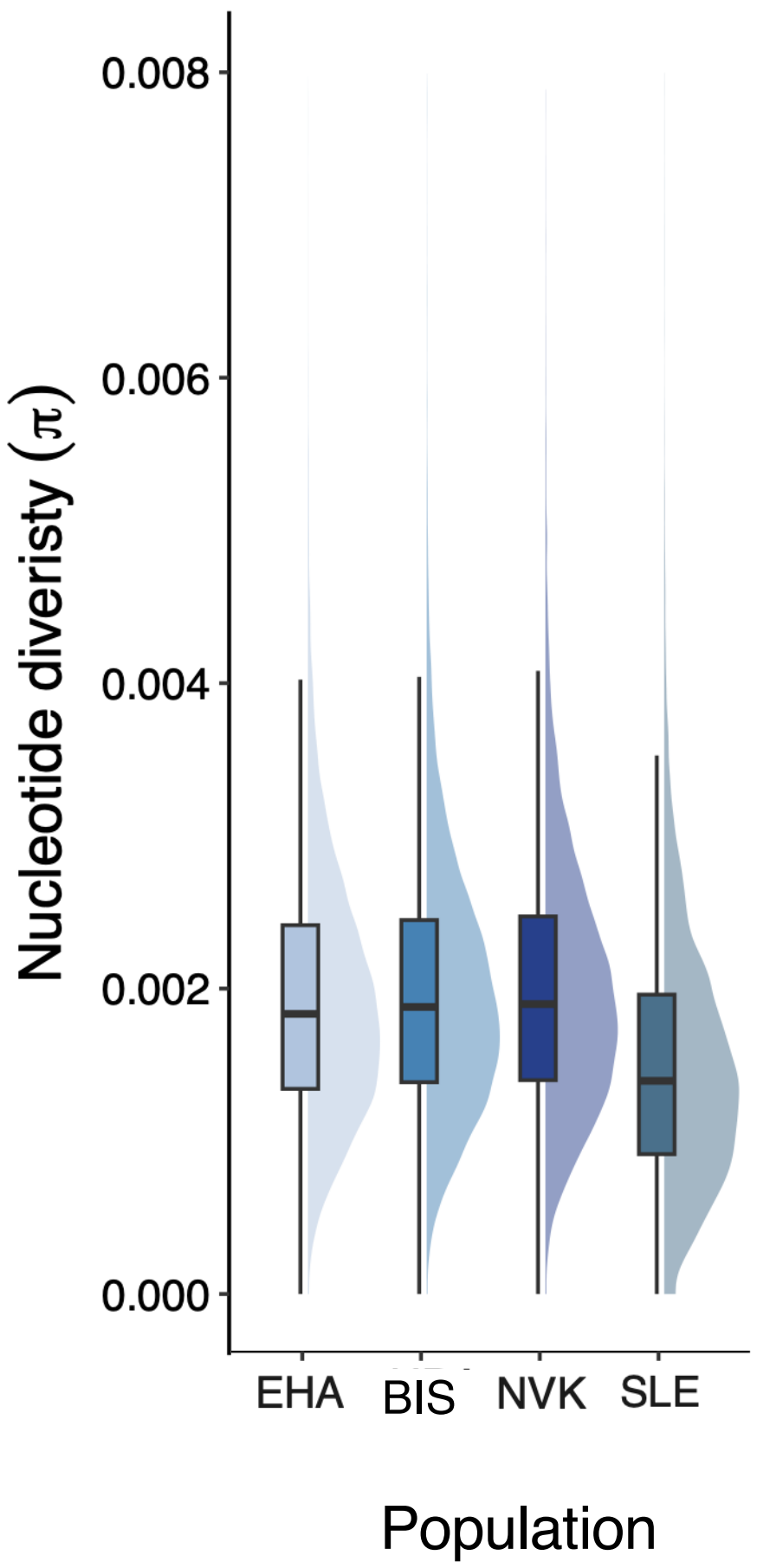

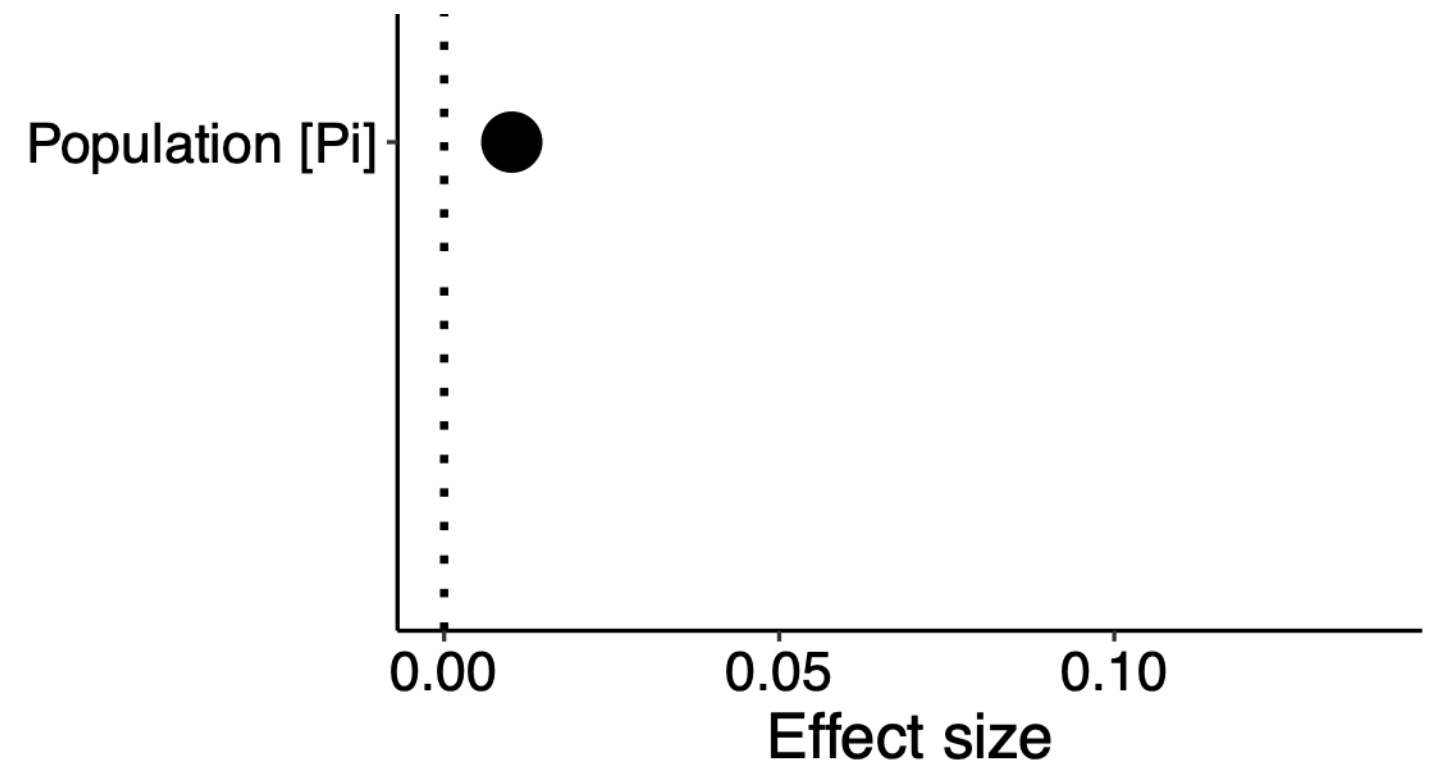

**Population**	**mean**	**2.5%**	**97.5%**
EHA	0.03	-0.02	0.08
BIS	0.05	-0.01	0.10
NVK	0.10	0.04	0.15
SLE	-0.17	-0.23	-0.12

Figure S4

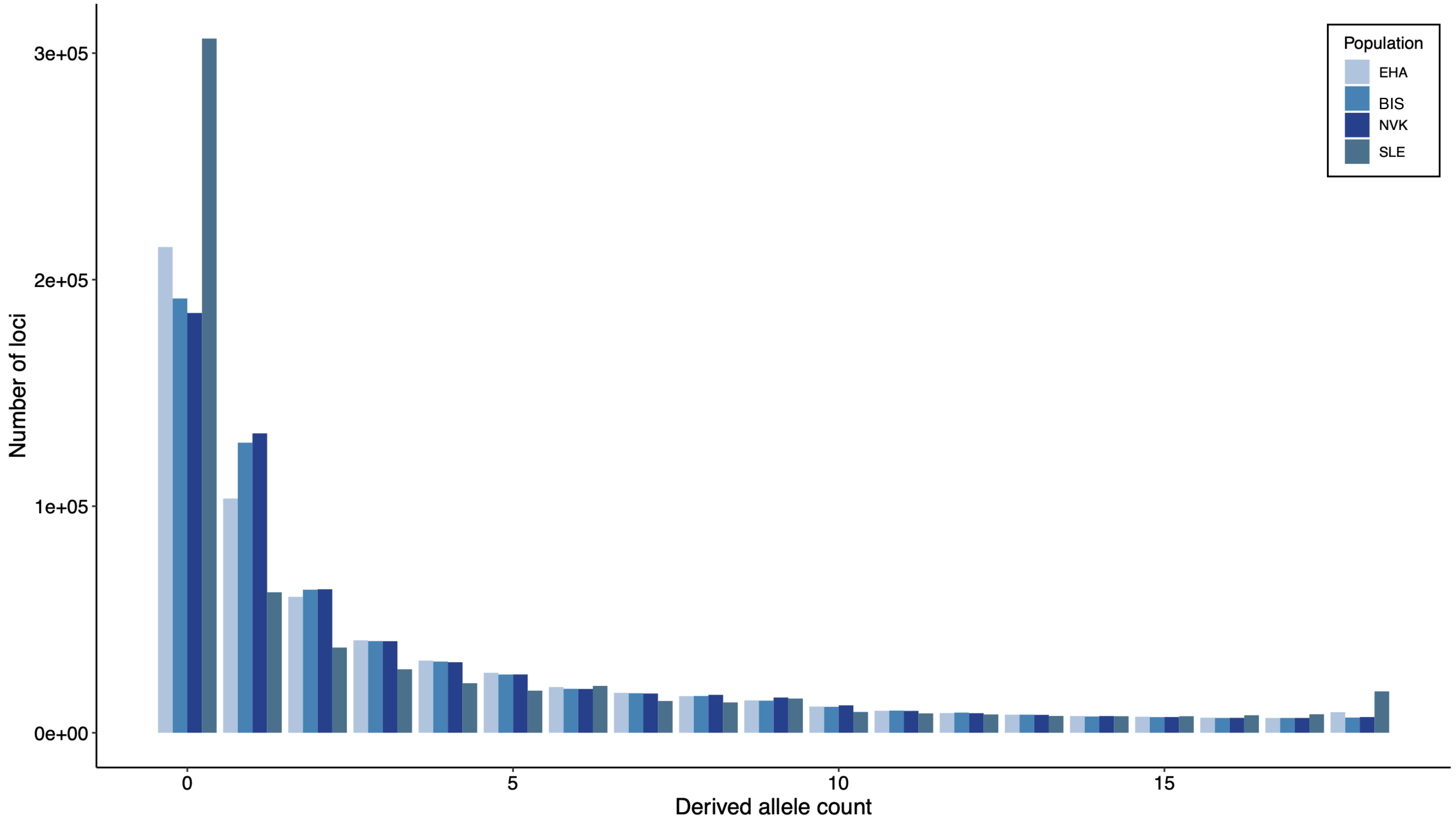

Population
EHA
BIS
NVK
SLE
3e+05
2e+05
1e+05
0e+00
Number of loci
0
5
10
15
Derived allele count

Figure S5

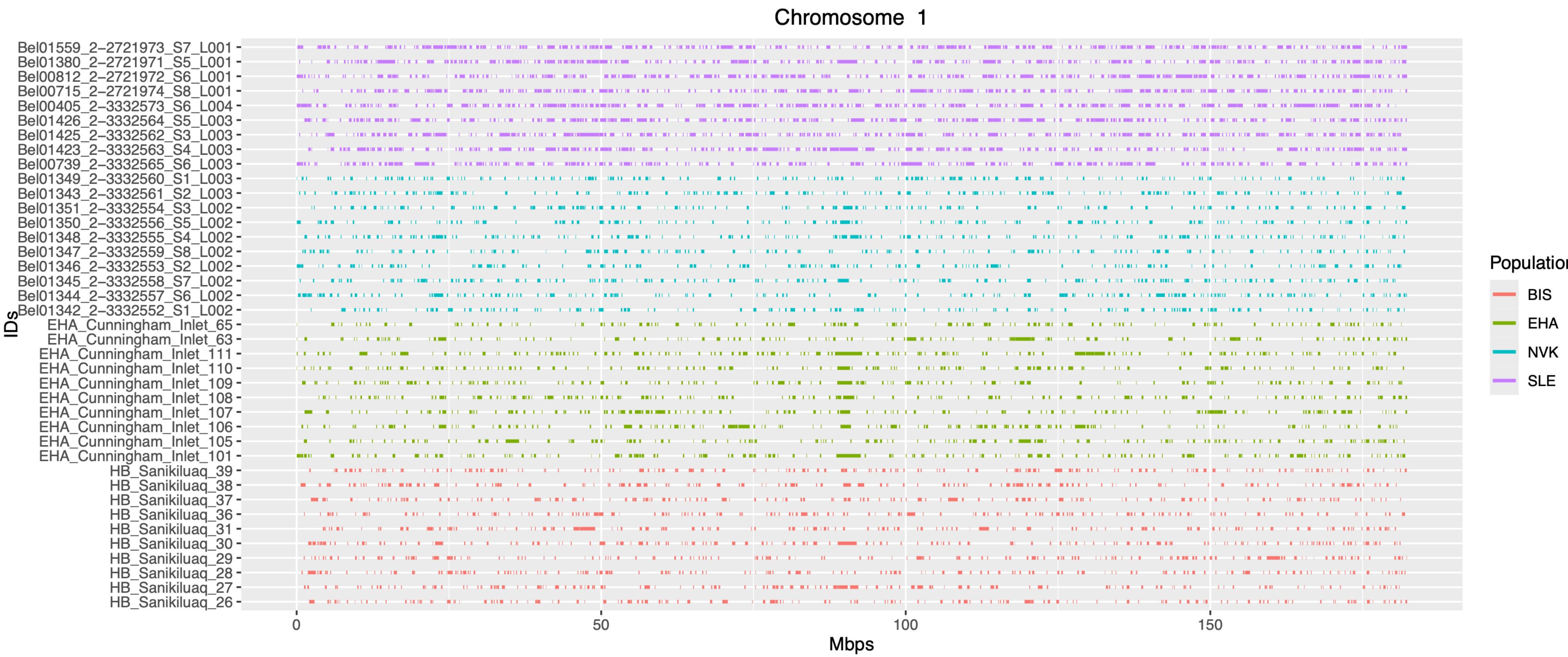

Chromosome 1
Bel01559_2-2721973_S7_L001
Bel01380_2-2721971_S5_L001
Bel00812_2-2721972_S6_L001
Bel00715_2-2721974_S8_L001
Bel00405_2-3332573_S6_L004
Bel01426_2-3332564_S5_L003
Bel01425_2-3332562_S3_L003
Bel01423_2-3332563_S4_L003
Bel00739_2-3332565_S6_L003
Bel01349_2-3332560_S1_L003
Bel01343_2-3332561_S2_L003
Bel01351_2-3332554_S3_L002
Bel01350_2-3332556_S5_L002
Bel01348_2-3332555_S4_L002
Bel01347_2-3332559_S8_L002
Bel01346_2-3332553_S2_L002
Bel01345_2-3332558_S7_L002
Bel01344_2-3332557_S6_L002
Bel01342_2-3332552_S1_L002
EHA_Cunningham_Inlet_65
EHA_Cunningham_Inlet_63
EHA_Cunningham_Inlet_111
EHA_Cunningham_Inlet_110
EHA_Cunningham_Inlet_109
EHA_Cunningham_Inlet_108
EHA_Cunningham_Inlet_107
EHA_Cunningham_Inlet_106
EHA_Cunningham_Inlet_105
EHA_Cunningham_Inlet_101
HB_Sanikiluaq_39
HB_Sanikiluaq_38
HB_Sanikiluaq_37
HB_Sanikiluaq_36
HB_Sanikiluaq_31
HB_Sanikiluaq_30
HB_Sanikiluaq_29
HB_Sanikiluaq_28
HB_Sanikiluaq_27
HB_Sanikiluaq_26
IDs
0
50
100
150
Mbps
Population
BIS
EHA
NVK
SLE

Figure S6

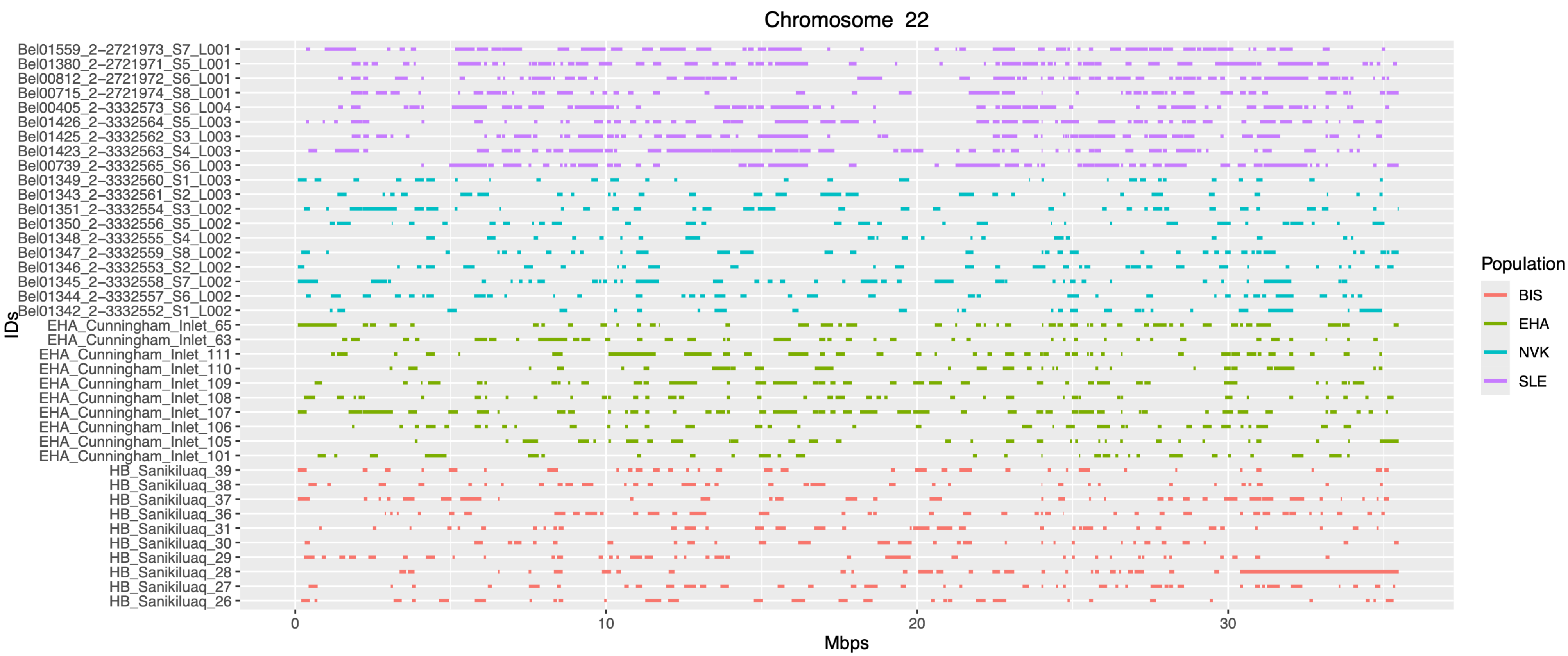

Chromosome 22
Bel01559_2-2721973_S7_L001
Bel01380_2-2721971_S5_L001
Bel00812_2-2721972_S6_L001
Bel00715_2-2721974_S8_L001
Bel00405_2-3332573_S6_L004
Bel01426_2-3332564_S5_L003
Bel01425_2-3332562_S3_L003
Bel01423_2-3332563_S4_L003
Bel00739_2-3332565_S6_L003
Bel01349_2-3332560_S1_L003
Bel01343_2-3332561_S2_L003
Bel01351_2-3332554_S3_L002
Bel01350_2-3332556_S5_L002
Bel01348_2-3332555_S4_L002
Bel01347_2-3332559_S8_L002
Bel01346_2-3332553_S2_L002
Bel01345_2-3332558_S7_L002
Bel01344_2-3332557_S6_L002
Bel01342_2-3332552_S1_L002
EHA_Cunningham_Inlet_65
EHA_Cunningham_Inlet_63
EHA_Cunningham_Inlet_111
EHA_Cunningham_Inlet_110
EHA_Cunningham_Inlet_109
EHA_Cunningham_Inlet_108
EHA_Cunningham_Inlet_107
EHA_Cunningham_Inlet_106
EHA_Cunningham_Inlet_105
EHA_Cunningham_Inlet_101
HB_Sanikiluaq_39
HB_Sanikiluaq_38
HB_Sanikiluaq_37
HB_Sanikiluaq_36
HB_Sanikiluaq_31
HB_Sanikiluaq_30
HB_Sanikiluaq_29
HB_Sanikiluaq_28
HB_Sanikiluaq_27
HB_Sanikiluaq_26
IDs
0
10
20
30
Mbps
Population
BIS
EHA
NVK
SLE

Figure S7

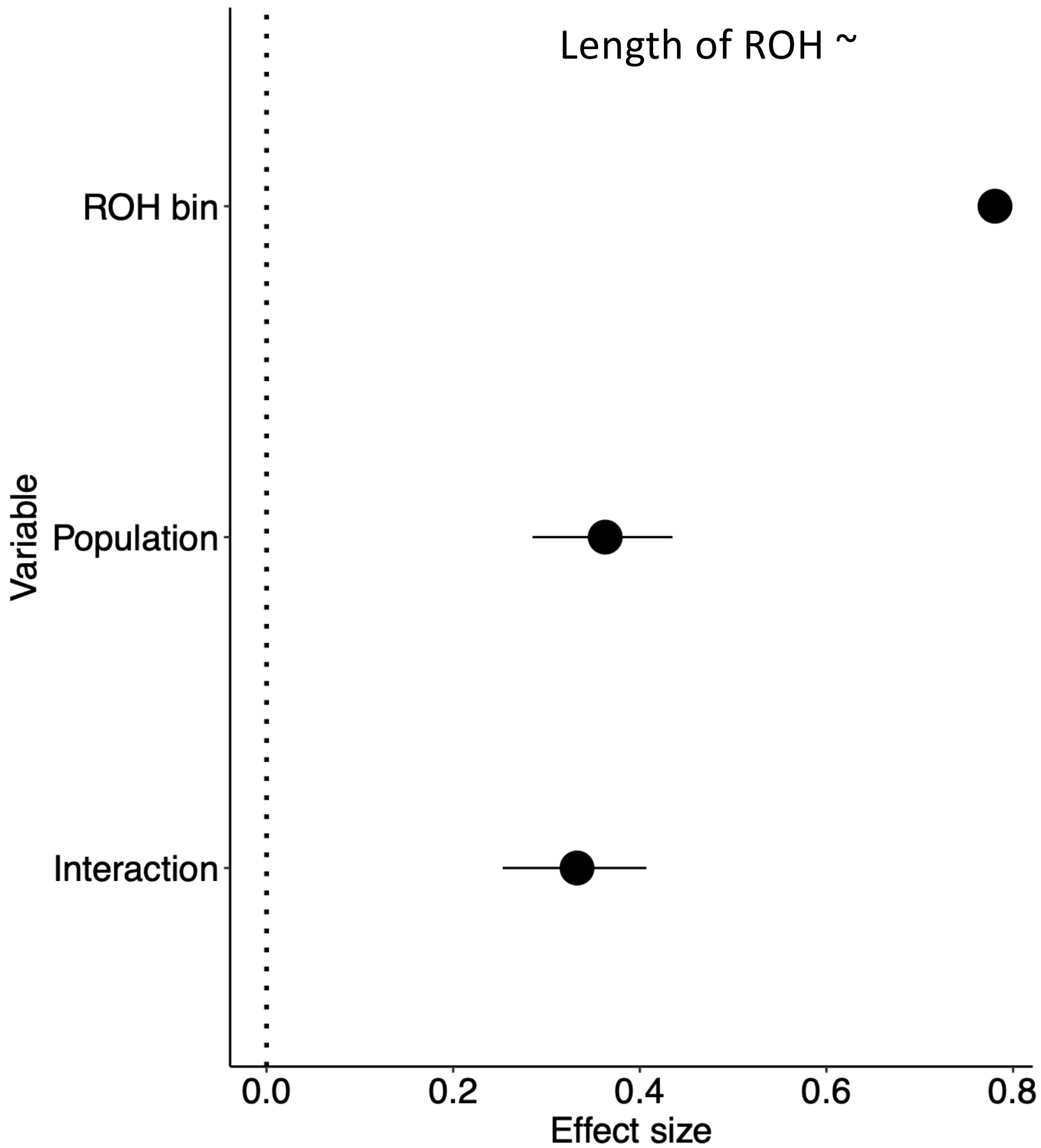

Population	mean	2.5%	97.5%
EHA	4.84	4.78	4.89
BIS	4.13	4.00	4.26
NVK	4.11	3.94	4.29
SLE	4.79	4.74	4.85

ROH bin	mean	2.5%	97.5%
<100Kb	-0.66	-0.67	-0.65
100Kb - 500K	-0.17	-0.18	-0.17
500Kb - 1Mb	1.26	1.25	1.28
1Mb - 5Mb	4.09	4.06	4.12
5Mb	17.82	17.53	18.12

Population	ROH.bin	mean	2.5%	97.5%
EHA	< 100Kb	-0.66	-0.67	-0.64
BIS	< 100Kb	-0.66	-0.67	-0.64
NVK	< 100Kb	-0.66	-0.67	-0.64
SLE	< 100Kb	-0.66	-0.67	-0.64
EHA	100Kb -500Kb	-0.19	-0.19	-0.18
BIS	100Kb -500Kb	-0.20	-0.21	-0.19
NVK	100Kb -500Kb	-0.20	-0.21	-0.19
SLE	100Kb -500Kb	-0.11	-0.12	-0.11
EHA	500Kb -1Mb	1.28	1.25	1.31
HB	500Kb -1Mb	1.23	1.20	1.26
NVK	500Kb -1Mb	1.22	1.19	1.25
SLE	500Kb -1Mb	1.32	1.30	1.33
EHA	1Mb - 5Mb	4.45	4.40	4.50
BIS	1Mb - 5Mb	3.92	3.85	3.98
NVK	1Mb - 5Mb	3.87	3.80	3.93
SLE	1Mb - 5Mb	4.14	4.12	4.17
EHA	> 5Mb	19.30	19.02	19.56
BIS	> 5Mb	16.37	15.70	17.01
NVK	> 5Mb	16.31	15.45	17.22
SLE	> 5Mb	19.28	19.01	19.56

Figure S8

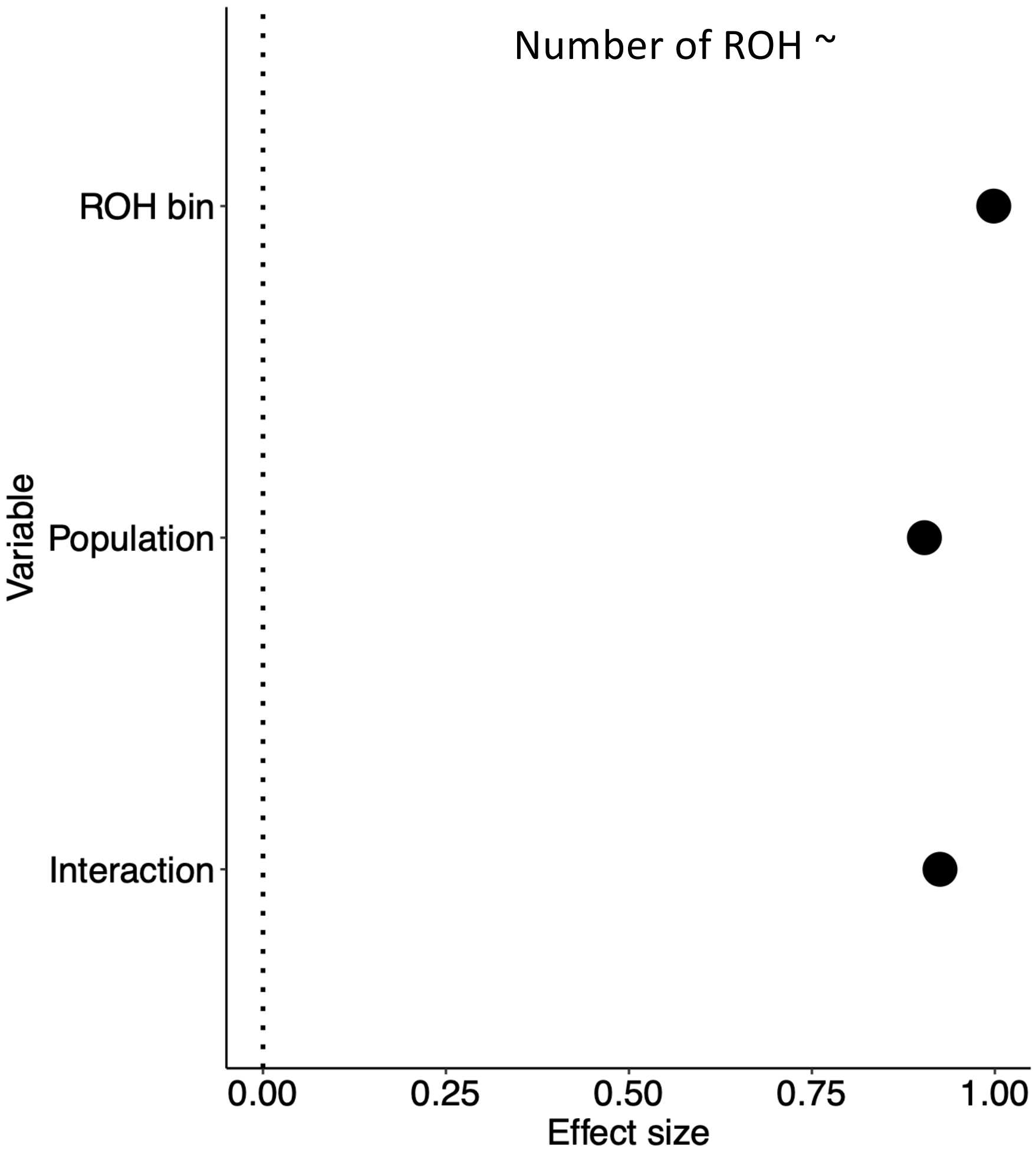

Population	mean	2.5%	97.5%
EHA	-0.17	-0.18	-0.15
BIS	-0.17	-0.18	-0.15
NVK	-0.16	-0.18	-0.14
SLE	0.12	0.10	0.13

ROH.bin	mean	2.5%	97.5%
< 100Kb	-0.28	-0.29	-0.27
100Kb - 500Kb	1.78	1.77	1.79
500Kb - 1Mb	-0.48	-0.50	-0.47
1Mb - 5Mb	-0.71	-0.72	-0.69
> 5Mb	-0.80	-0.82	-0.77

Population	ROH.bin	mean	2.5%	97.5%
EHA	< 100Kb	-0.28	-0.30	-0.25
HB	< 100Kb	-0.28	-0.31	-0.26
NVK	< 100Kb	-0.25	-0.27	-0.22
SLE	< 100Kb	-0.31	-0.34	-0.29
EHA	100Kb - 500Kb	1.54	1.52	1.57
HB	100Kb - 500Kb	1.60	1.57	1.62
NVK	100Kb - 500Kb	1.61	1.58	1.63
SLE	100Kb - 500Kb	2.38	2.35	2.40
EHA	500Kb - 1Mb	-0.59	-0.61	-0.56
HB	500Kb - 1Mb	-0.60	-0.63	-0.58
NVK	500Kb - 1Mb	-0.62	-0.64	-0.59
SLE	500Kb - 1Mb	-0.12	-0.15	-0.10
EHA	1Mb - 5Mb	-0.73	-0.76	-0.71
HB	1Mb - 5Mb	-0.76	-0.78	-0.74
NVK	1Mb - 5Mb	-0.76	-0.79	-0.74
SLE	1Mb - 5Mb	-0.57	-0.59	-0.54
EHA	> 5Mb	-0.79	-0.83	-0.75
HB	> 5Mb	-0.80	-0.85	-0.74
NVK	> 5Mb	-0.80	-0.87	-0.72
SLE	> 5Mb	-0.80	-0.83	-0.77

Figure S9

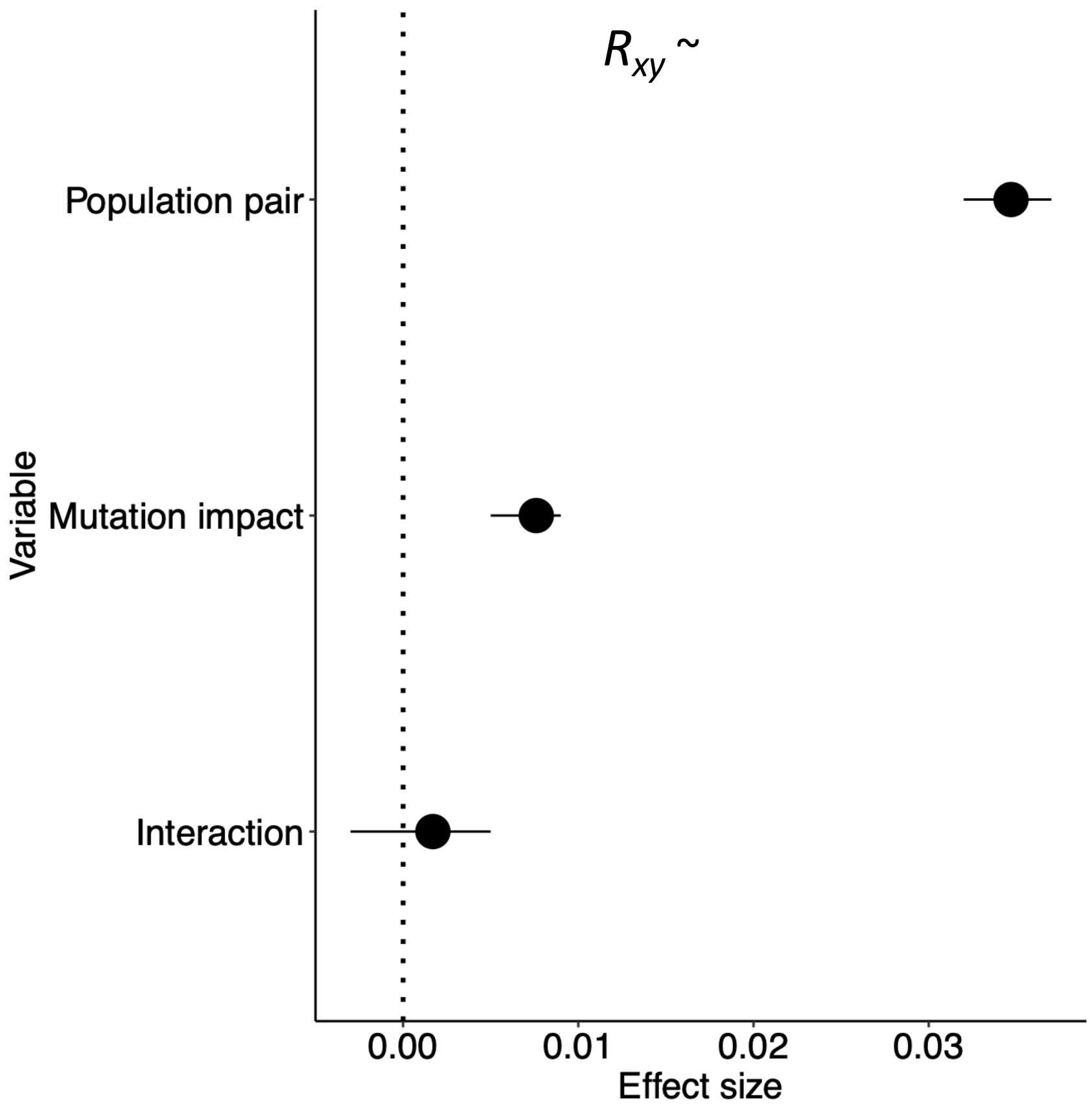

Impact	mean	2.5%	97.5%
High	0.15	0.07	0.22
Low	-0.11	-0.19	-0.04
Moderate	0.00	-0.07	0.08
Modifier	-0.02	-0.10	0.06

Population pair	mean	2.5%	97.5%
SLE_EHA	0.21	0.12	0.30
SLE_NVK	0.00	-0.10	0.10
SLE_BIS	0.24	0.15	0.33
EHA_BIS	0.04	-0.06	0.13
EHA_NVK	-0.22	-0.32	-0.12
EHB_NVK	-0.25	-0.35	-0.16

Figure S10

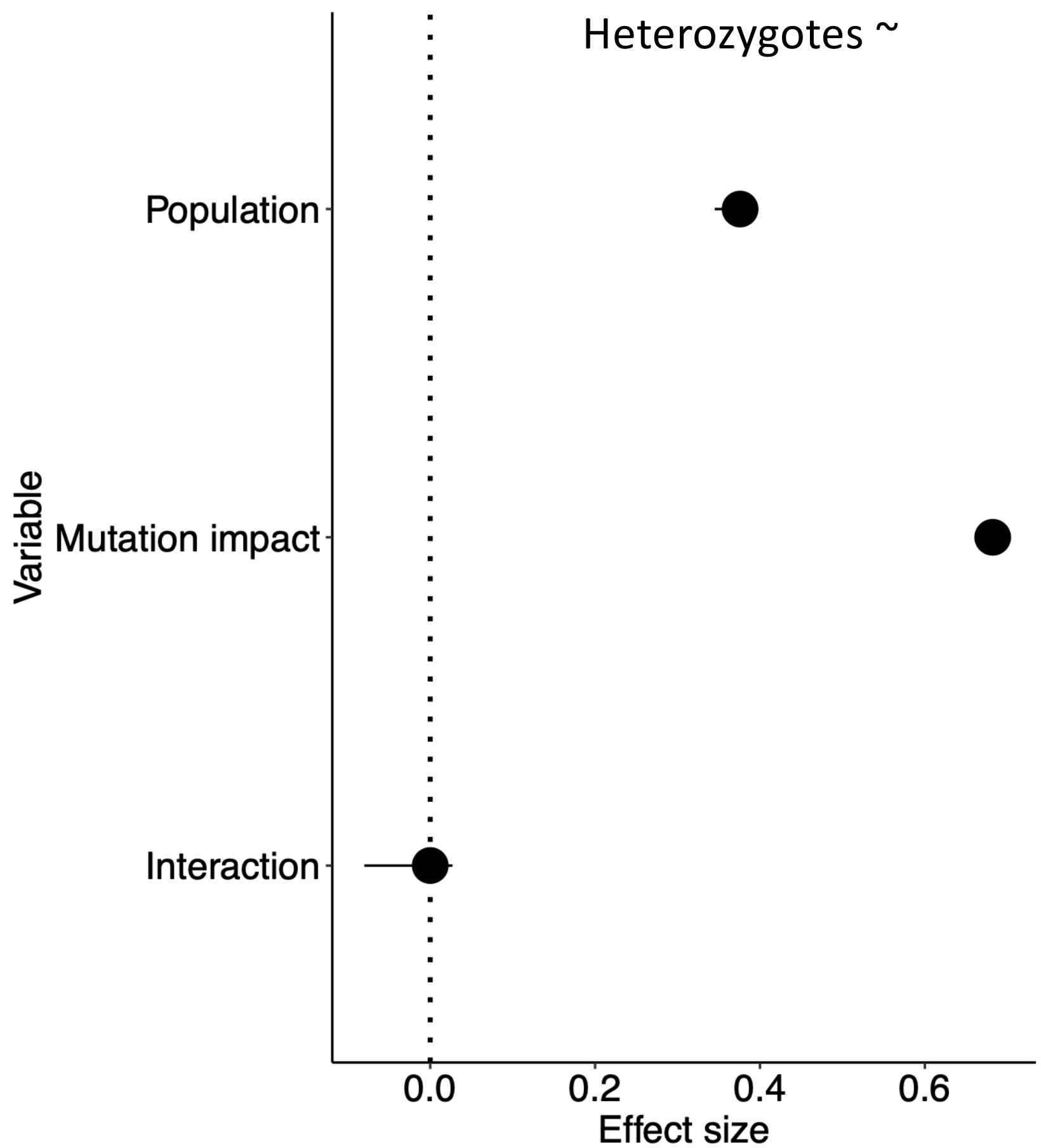

Impact	mean	2.5%	97.5%
Modifier	-0.93	-1.09	-0.78
Low	-0.52	-0.68	-0.35
Moderate	0.52	0.36	0.69
High	0.95	0.79	1.10

Population	mean	2.5%	97.5%
EHA	0.13	-0.04	0.28
BIS	0.10	-0.07	0.26
NVK	0.46	0.29	0.63
SLE	-0.66	-0.82	-0.49

Figure S11

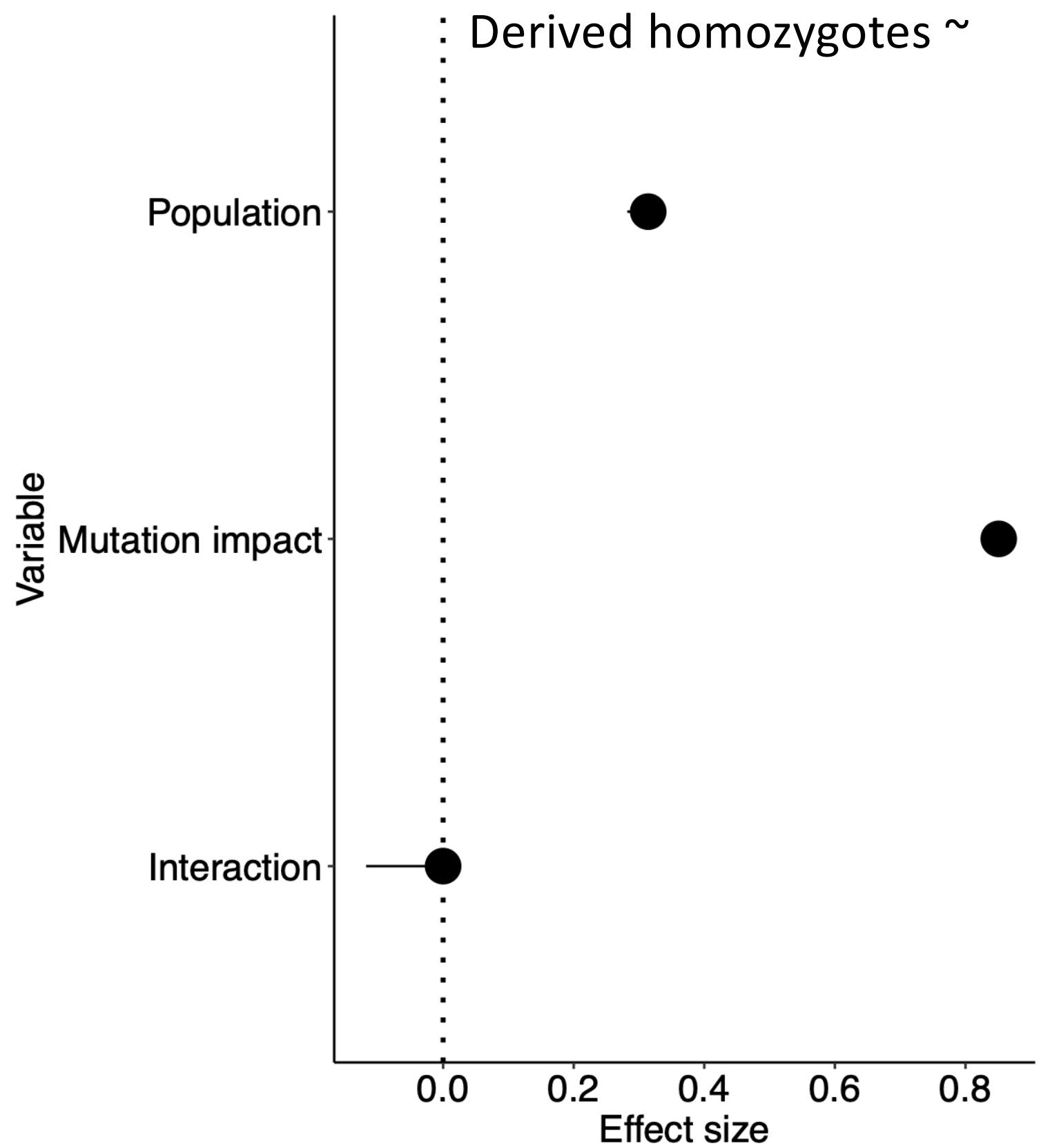

**Impact**	**mean**	**2.5%**	**97.5%**
Modifier	1.20	1.09	1.33
Low	0.46	0.34	0.56
Moderate	-0.55	-0.68	-0.44
High	-1.11	-1.22	-1.00

**Population**	**mean**	**2.5%**	**97.5%**
EHA	-0.12	-0.23	0.00
BIS	-0.11	-0.23	0.00
NVK	-0.22	-0.34	-0.09
SLE	0.44	0.33	0.56

Figure S12

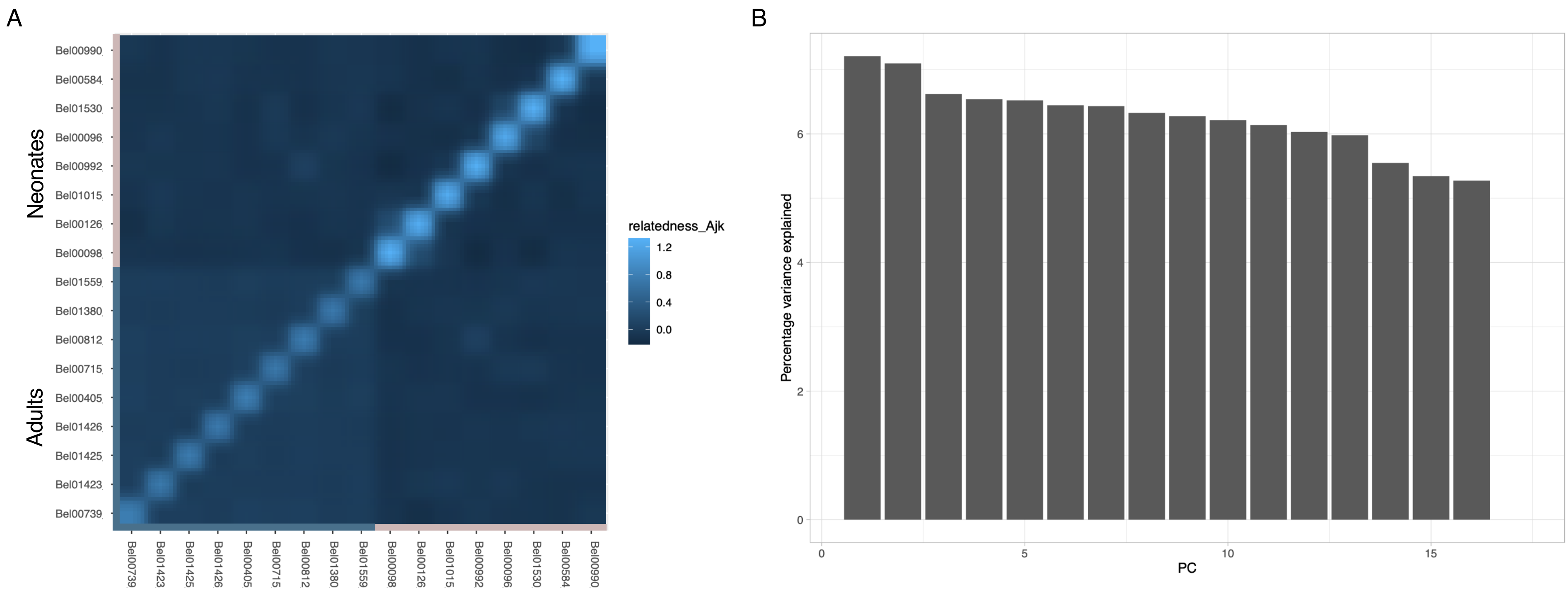
A
B
Neonates
Adults
Bel00990
Bel00584
Bel01530
Bel00096
Bel00992
Bel01015
Bel00126
Bel00098
Bel01559
Bel01380
Bel00812
Bel00715
Bel00405
Bel01426
Bel01425
Bel01423
Bel00739
relatedness_Ajk
1.2
0.8
0.4
0.0
Percentage variance explained
PC
0
2
4
6
5
10
15

Figure S13

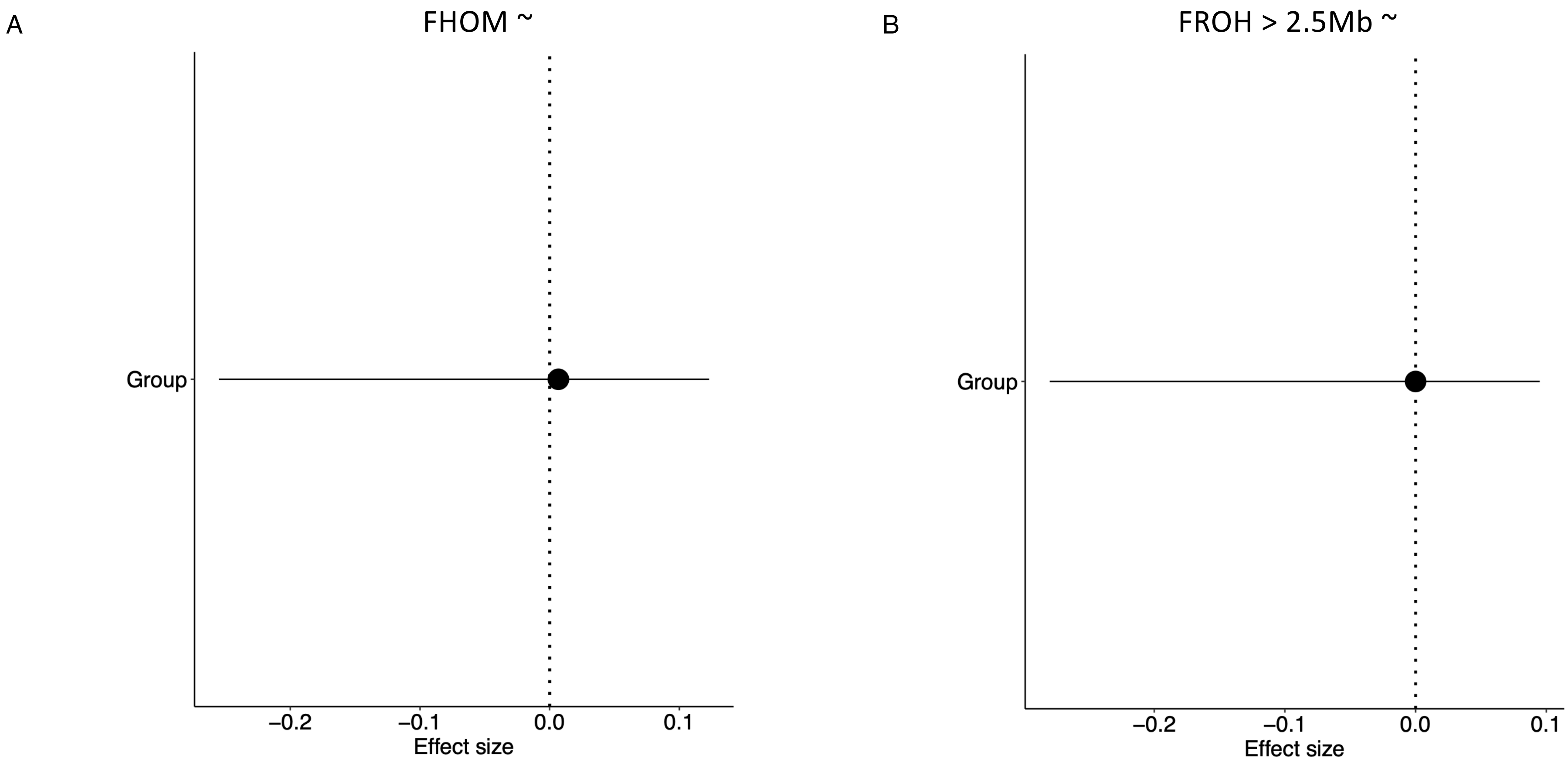
A
FHOM ~
Group
−0.2
−0.1
0.0
0.1
Effect size
B
FROH > 2.5Mb ~
Group
−0.2
−0.1
0.0
0.1
Effect size

Figure S14

A

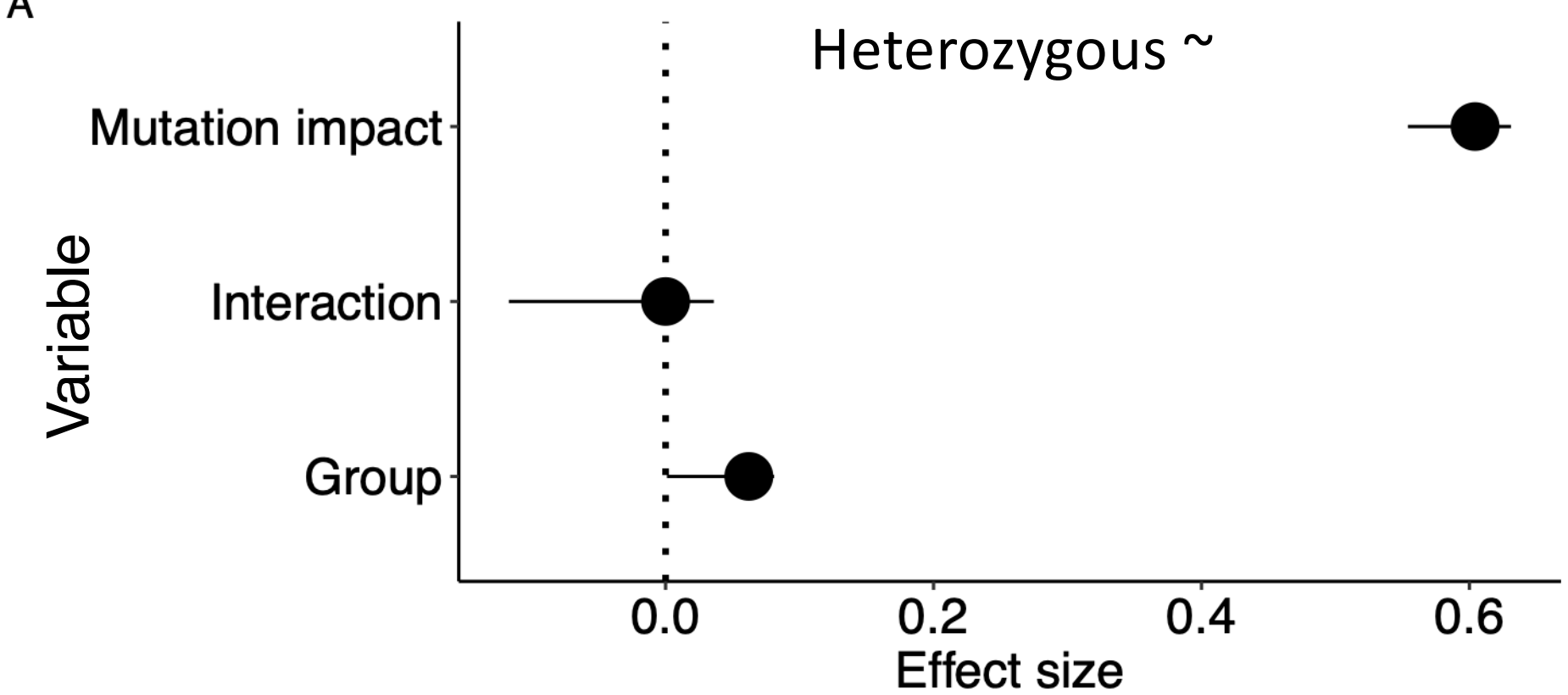

Impact	mean	2.5%	97.5%
High	1.28	0.96	1.59
Low	-0.52	-0.80	-0.22
Moderate	-0.14	-0.46	0.18
Modifier	-0.67	-0.99	-0.36

Group	mean	2.5%	97.5%
Adult	0.16	-0.04	0.37
Neonate	-0.19	-0.41	0.04

B

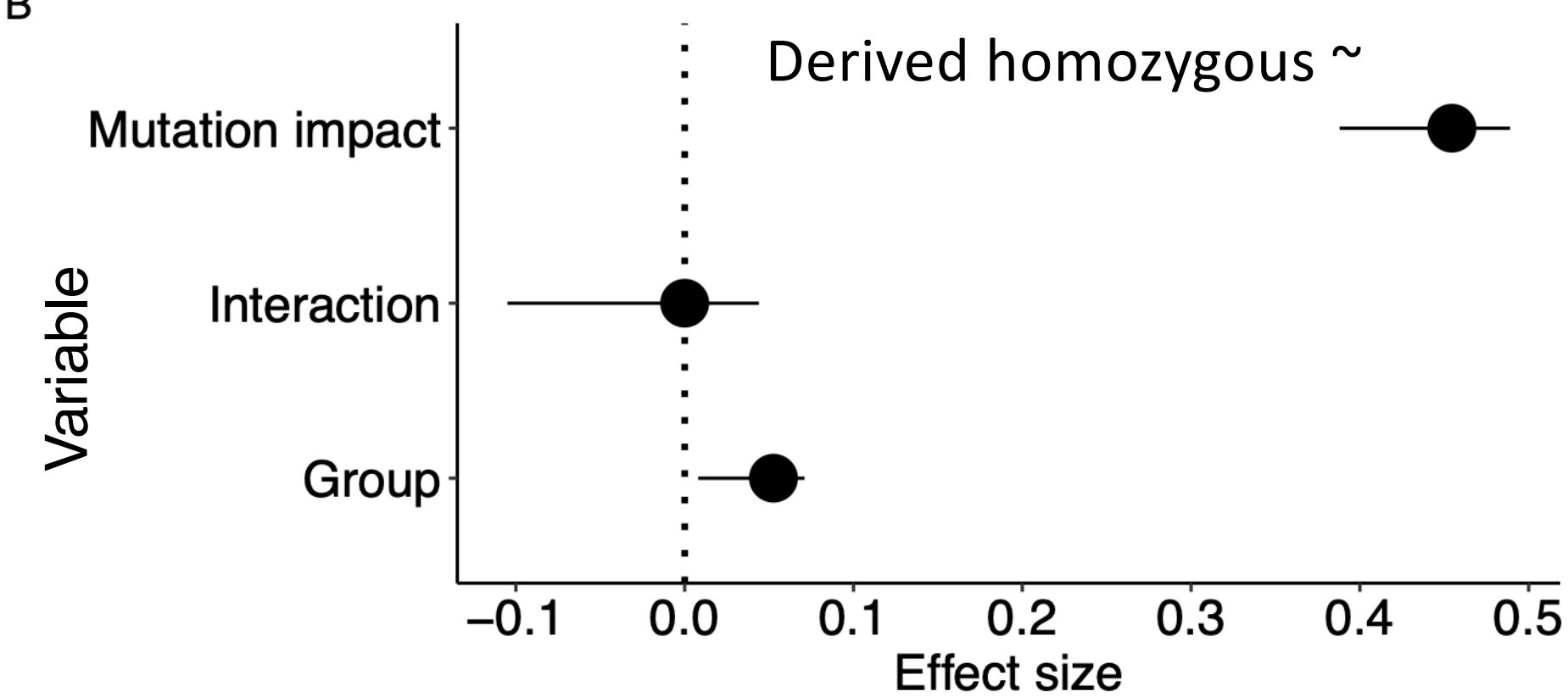

Impact	mean	2.5%	97.5%
High	-1.02	-1.21	-0.83
Low	0.56	0.38	0.75
Moderate	-0.76	-0.94	-0.57
Modifier	1.23	1.04	1.41

Group	mean	2.5%	97.5%
Adult	-0.04	-0.17	0.09
Neonate	0.04	-0.09	0.18

Figure S15

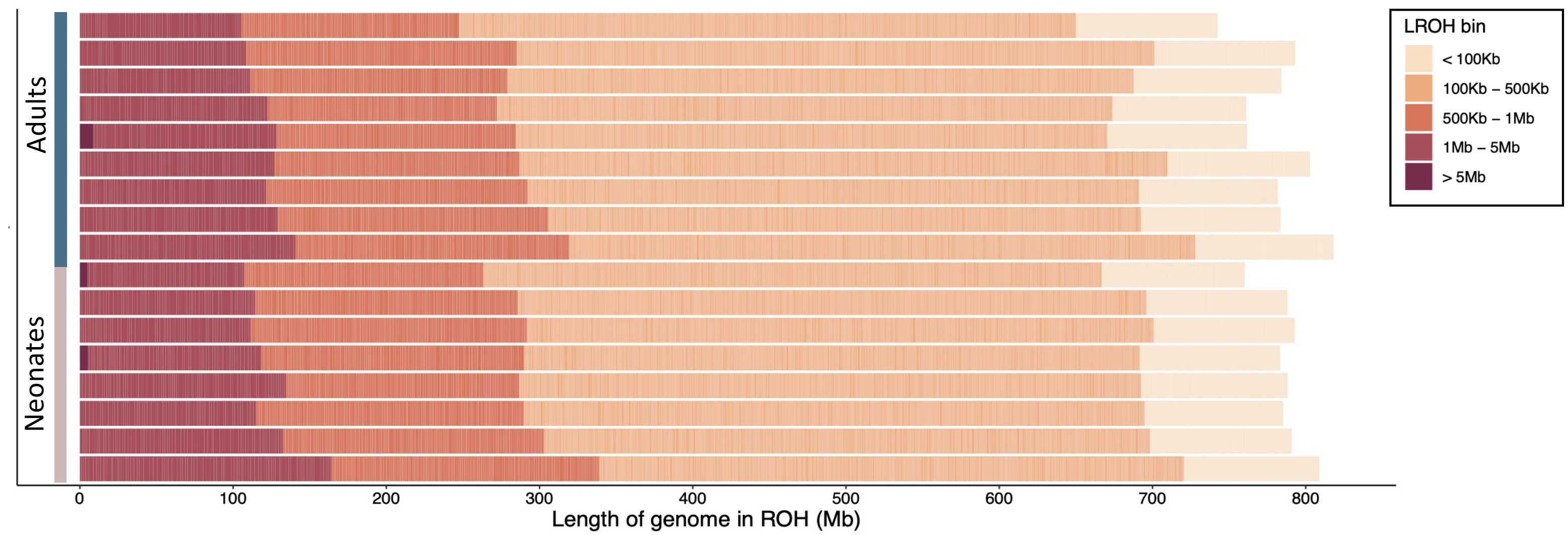
Adults
Neonates
LROH bin
< 100Kb
100Kb – 500Kb
500Kb – 1Mb
1Mb – 5Mb
> 5Mb
0
100
200
300
400
500
600
700
800
Length of genome in ROH (Mb)

Figure S16

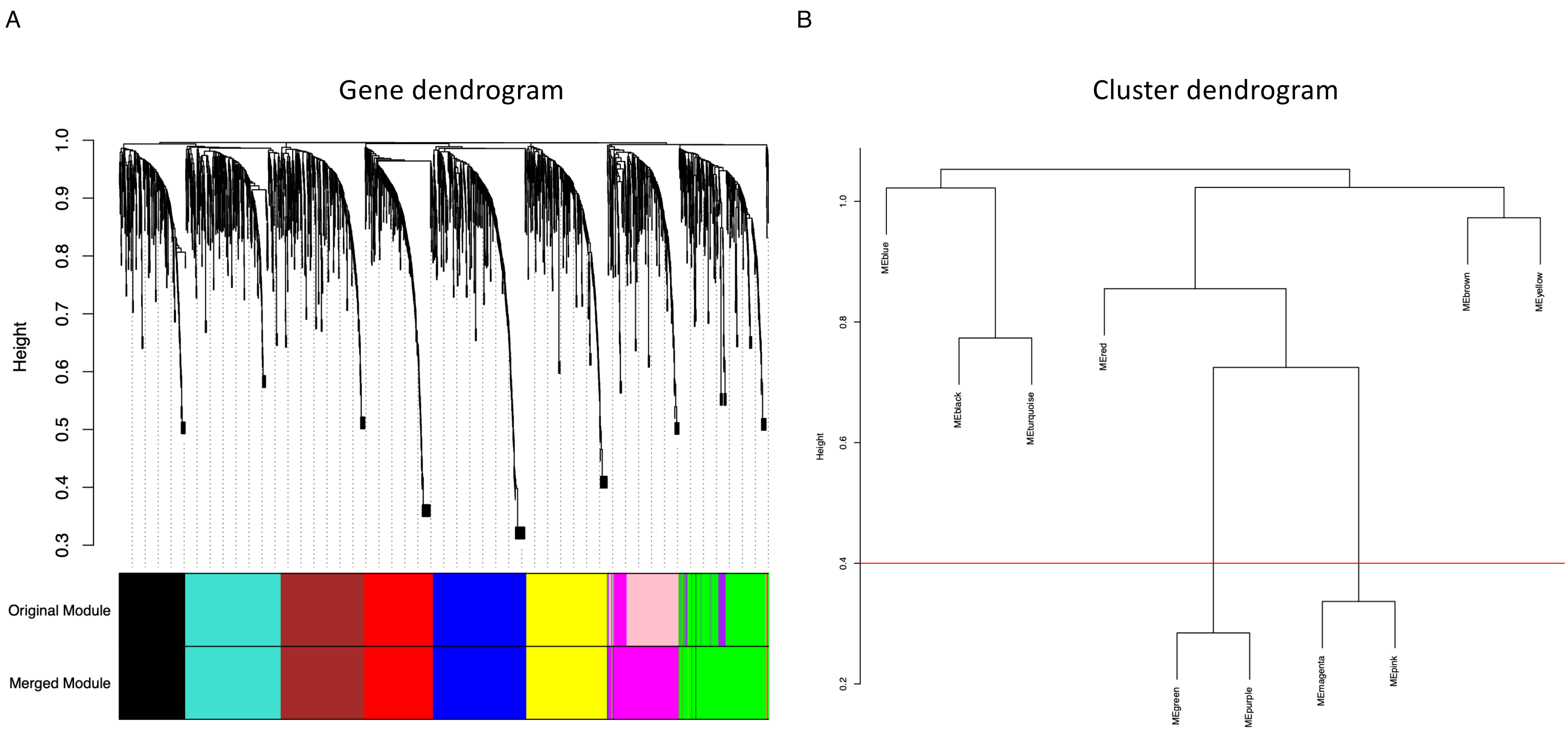
A
Gene dendrogram
Height
1.0
0.9
0.8
0.7
0.6
0.5
0.4
0.3
Original Module
Merged Module
B
Cluster dendrogram
Height
1.0
0.8
0.6
0.4
0.2
MEblue
MEblack
MEturquoise
MEred
MEgreen
MEpurple
MEmagenta
MEpink
MEbrown
MEyellow

Figure S17

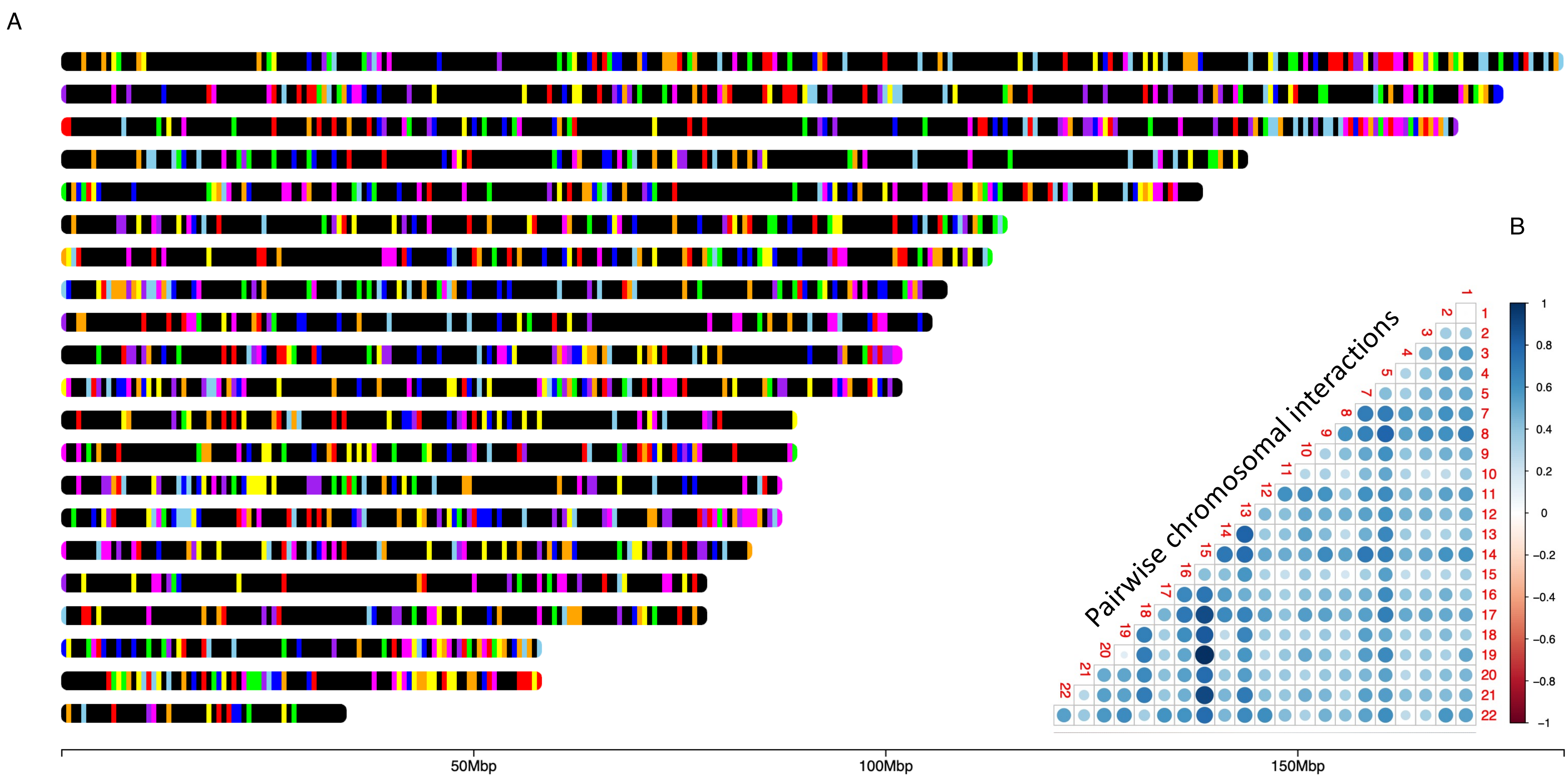
A
B
50Mbp
100Mbp
150Mbp
Pairwise chromosomal interactions
1
0.8
0.6
0.4
0.2
0
−0.2
−0.4
−0.6
−0.8
−1

Figure S18

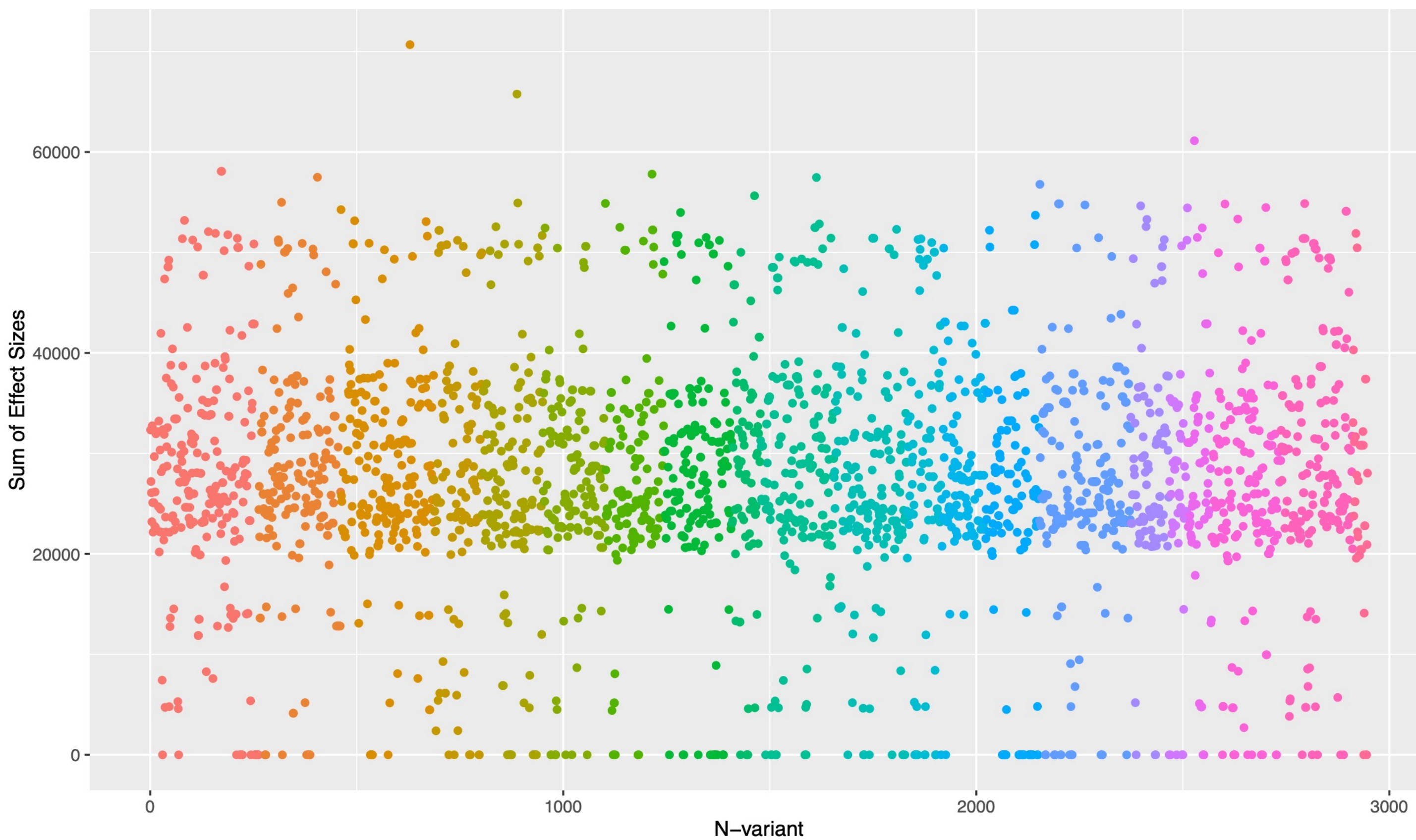
60000
40000
20000
0
Sum of Effect Sizes
0
1000
2000
3000
N−variant

Figure S19

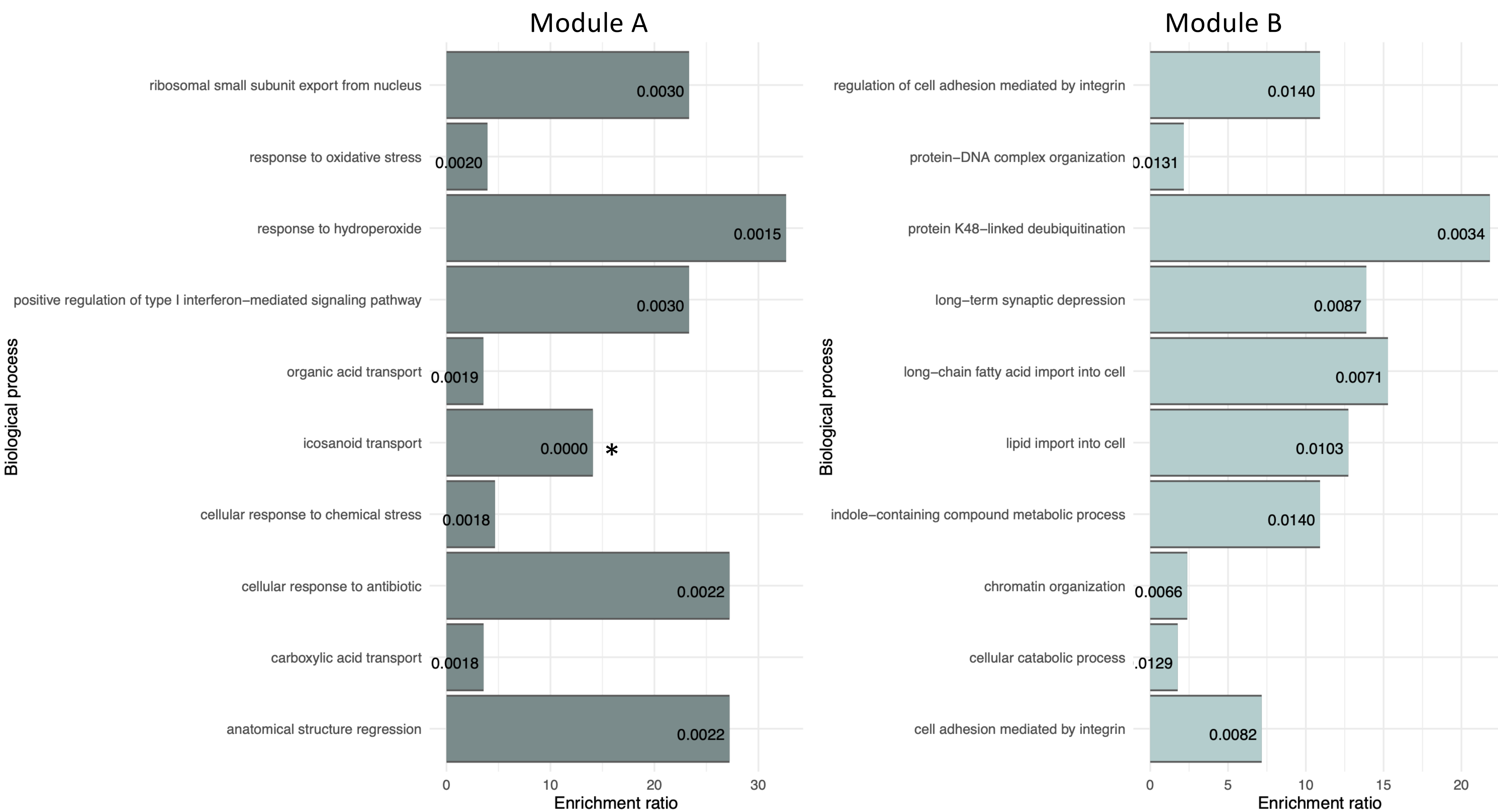

Module A
ribosomal small subunit export from nucleus
response to oxidative stress
response to hydroperoxide
positive regulation of type I interferon−mediated signaling pathway
organic acid transport
icosanoid transport
cellular response to chemical stress
cellular response to antibiotic
carboxylic acid transport
anatomical structure regression
0.0030
0.0020
0.0015
0.0030
0.0019
0.0000
*
0.0018
0.0022
0.0018
0.0022
Biological process
0
10
20
30
Enrichment ratio
Module B
regulation of cell adhesion mediated by integrin
protein−DNA complex organization
protein K48−linked deubiquitination
long−term synaptic depression
long−chain fatty acid import into cell
lipid import into cell
indole−containing compound metabolic process
chromatin organization
cellular catabolic process
cell adhesion mediated by integrin
0.0140
0.0131
0.0034
0.0087
0.0071
0.0103
0.0140
0.0066
.0129
0.0082
Biological process
0
5
10
15
20
Enrichment ratio

Figure S20

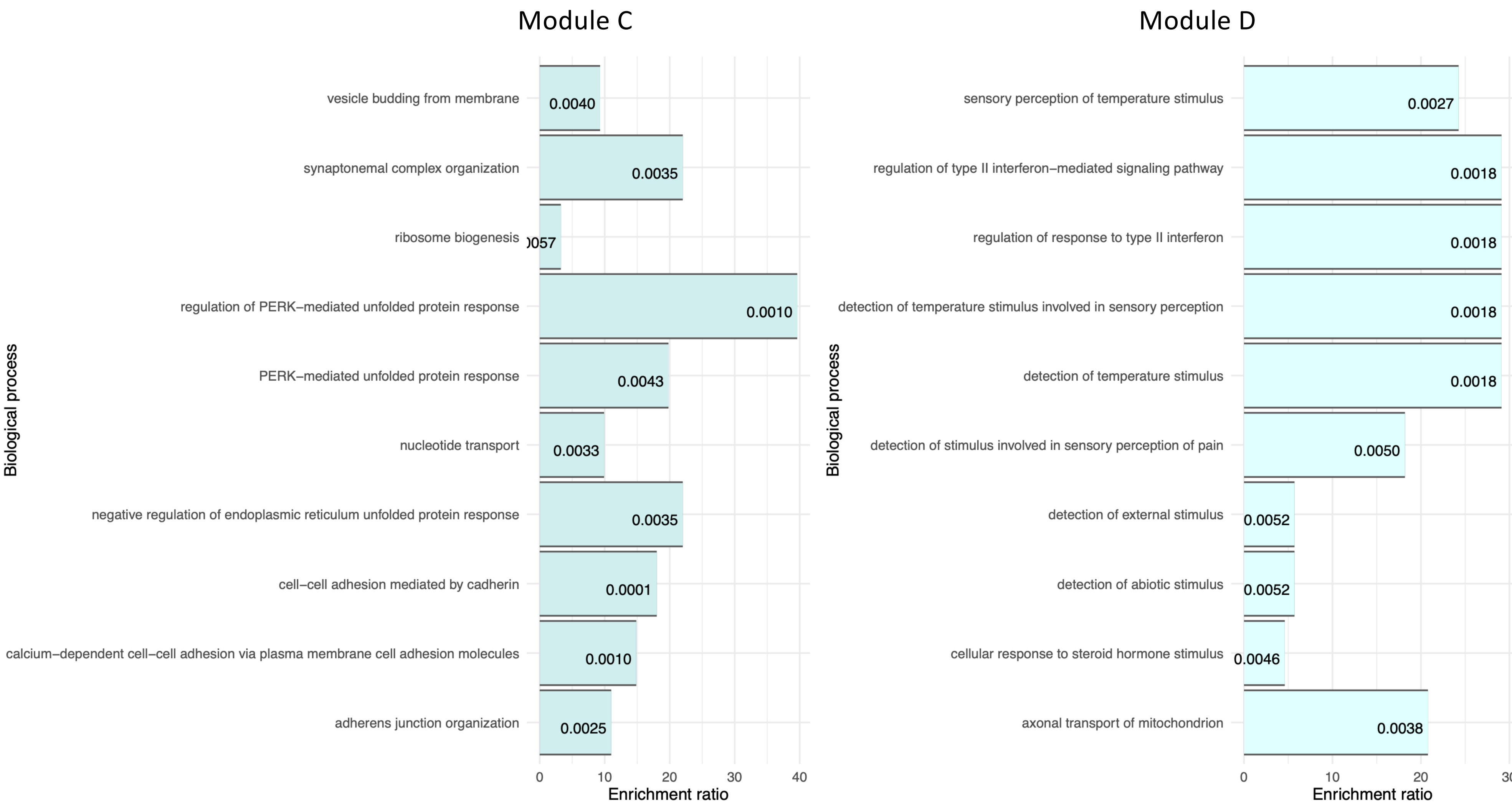

Module C
vesicle budding from membrane
0.0040
synaptonemal complex organization
0.0035
ribosome biogenesis
)057
regulation of PERK−mediated unfolded protein response
0.0010
PERK−mediated unfolded protein response
0.0043
nucleotide transport
0.0033
negative regulation of endoplasmic reticulum unfolded protein response
0.0035
cell−cell adhesion mediated by cadherin
0.0001
calcium−dependent cell−cell adhesion via plasma membrane cell adhesion molecules
0.0010
adherens junction organization
0.0025
Biological process
0
10
20
30
40
Enrichment ratio
Module D
sensory perception of temperature stimulus
0.0027
regulation of type II interferon−mediated signaling pathway
0.0018
regulation of response to type II interferon
0.0018
detection of temperature stimulus involved in sensory perception
0.0018
detection of temperature stimulus
0.0018
detection of stimulus involved in sensory perception of pain
0.0050
detection of external stimulus
0.0052
detection of abiotic stimulus
0.0052
cellular response to steroid hormone stimulus
0.0046
axonal transport of mitochondrion
0.0038
Biological process
0
10
20
30
Enrichment ratio

Figure S21

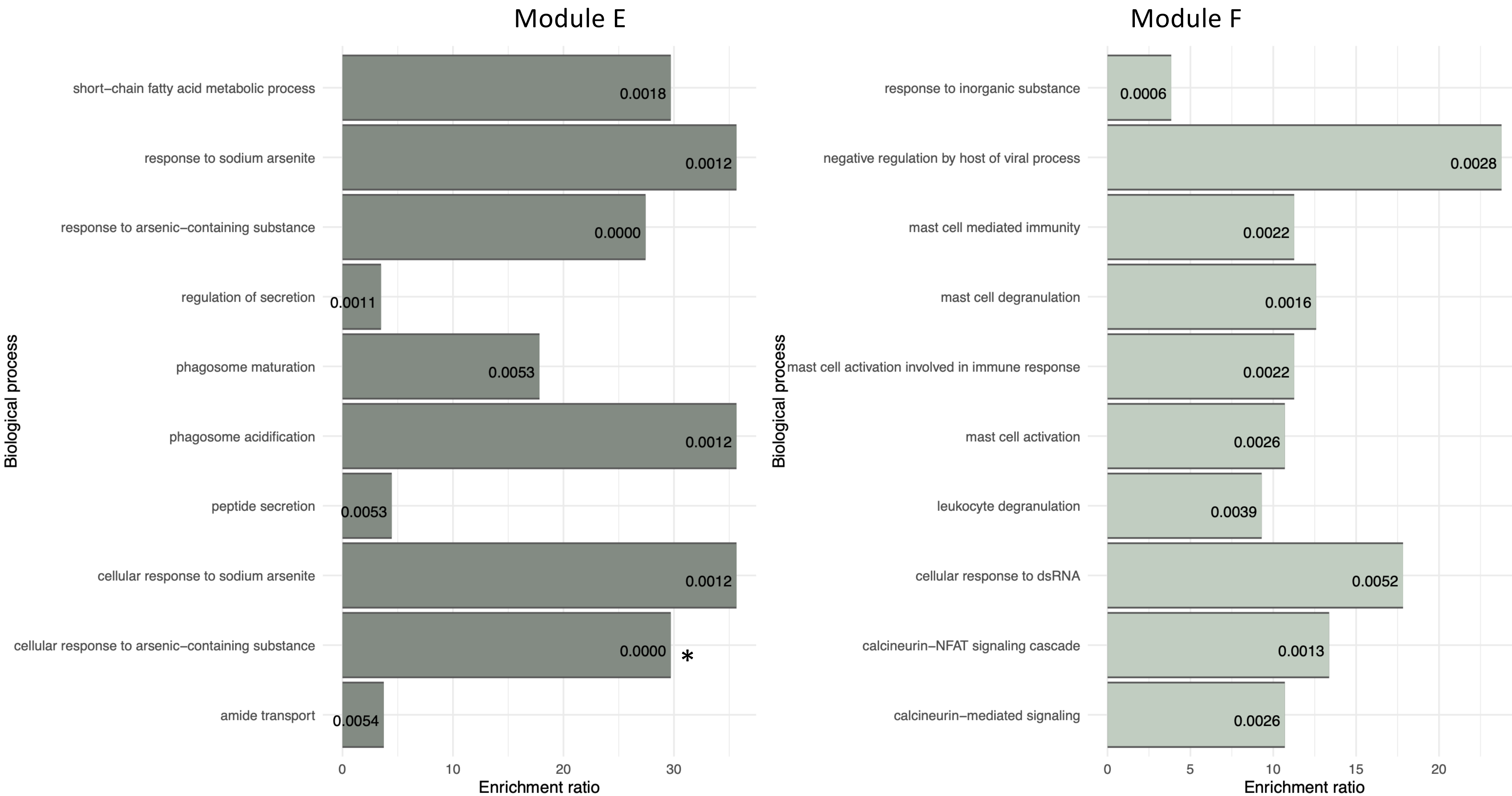

Module E
short−chain fatty acid metabolic process
response to sodium arsenite
response to arsenic−containing substance
regulation of secretion
phagosome maturation
phagosome acidification
peptide secretion
cellular response to sodium arsenite
cellular response to arsenic−containing substance
amide transport
0.0018
0.0012
0.0000
0.0011
0.0053
0.0012
0.0053
0.0012
0.0000
*
0.0054
Biological process
0
10
20
30
Enrichment ratio
Module F
response to inorganic substance
negative regulation by host of viral process
mast cell mediated immunity
mast cell degranulation
mast cell activation involved in immune response
mast cell activation
leukocyte degranulation
cellular response to dsRNA
calcineurin−NFAT signaling cascade
calcineurin−mediated signaling
0.0006
0.0028
0.0022
0.0016
0.0022
0.0026
0.0039
0.0052
0.0013
0.0026
Biological process
0
5
10
15
20
Enrichment ratio

Figure S22

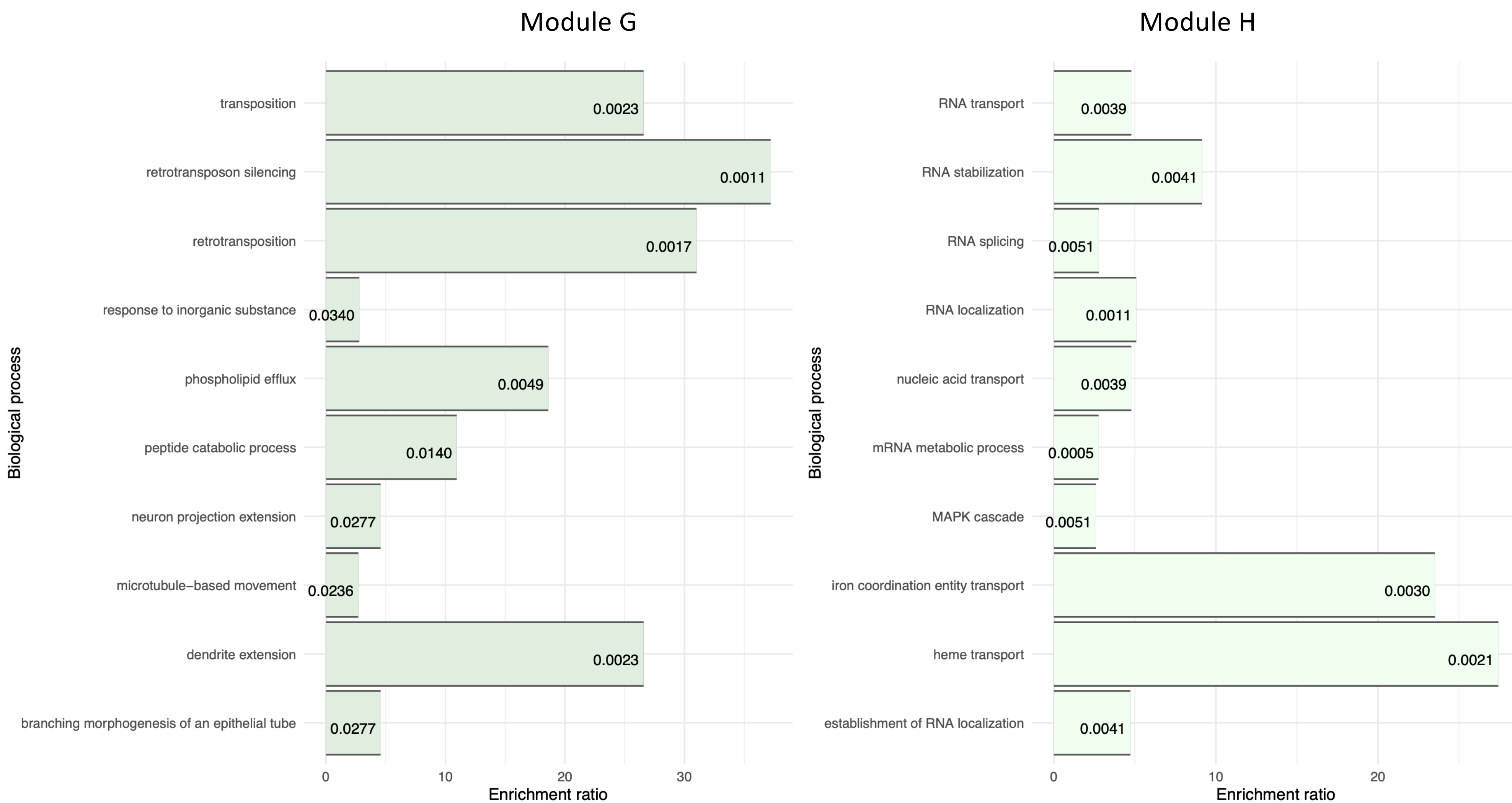
Module G
transposition
retrotransposon silencing
retrotransposition
response to inorganic substance
phospholipid efflux
peptide catabolic process
neuron projection extension
microtubule−based movement
dendrite extension
branching morphogenesis of an epithelial tube
0.0023
0.0011
0.0017
0.0340
0.0049
0.0140
0.0277
0.0236
0.0023
0.0277
Biological process
0
10
20
30
Enrichment ratio
Module H
RNA transport
RNA stabilization
RNA splicing
RNA localization
nucleic acid transport
mRNA metabolic process
MAPK cascade
iron coordination entity transport
heme transport
establishment of RNA localization
0.0039
0.0041
0.0051
0.0011
0.0039
0.0005
0.0051
0.0030
0.0021
0.0041
Biological process
0
10
20
Enrichment ratio

Figure S23

Sample ID	Site	Group	Sampling year	Sex	Coverage
EHA_Cunningham_Inlet_101	Cunningham Inlet	EHA	1996	female	12.68
EHA_Cunningham_Inlet_105	Cunningham Inlet	EHA	1991	female	14.98
EHA_Cunningham_Inlet_106	Cunningham Inlet	EHA	1996	female	12.58
EHA_Cunningham_Inlet_107	Cunningham Inlet	EHA	1999	female	10.06
EHA_Cunningham_Inlet_108	Cunningham Inlet	EHA	1999	male	11.11
EHA_Cunningham_Inlet_109	Cunningham Inlet	EHA	1999	female	11.34
EHA_Cunningham_Inlet_110	Cunningham Inlet	EHA	1999	male	13.34
EHA_Cunningham_Inlet_111	Cunningham Inlet	EHA	1999	male	13.60
EHA_Cunningham_Inlet_63	Cunningham Inlet	EHA	1999	male	12.38
EHA_Cunningham_Inlet_65	Cunningham Inlet	EHA	1999	male	15.84
Bel01342	Nunavik	NVK	2014	male	18.63
Bel01343	Nunavik	NVK	2014	male	17.40
Bel01344	Nunavik	NVK	2014	male	29.13
Bel01345	Nunavik	NVK	2014	female	22.35
Bel01346	Nunavik	NVK	2014	male	22.42
Bel01347	Nunavik	NVK	2014	male	33.88
Bel01348	Nunavik	NVK	2014	female	30.75
Bel01349	Nunavik	NVK	2014	female	30.24
Bel01350	Nunavik	NVK	2014	male	22.49
Bel01351	Nunavik	NVK	2014	male	9.59
Bel00096	Saint Lawrence	neonate_mortality	1995	male	18.50
Bel00098	Saint Lawrence	neonate_mortality	1995	male	26.66
Bel00126	Saint Lawrence	neonate_mortality	1996	female	39.16
Bel00584	Saint Lawrence	neonate_mortality	2007	female	20.27
Bel00990	Saint Lawrence	neonate_mortality	2013	female	20.42
Bel00992	Saint Lawrence	neonate_mortality	2013	female	21.50
Bel01015	Saint Lawrence	neonate_mortality	2014	female	17.38
Bel01530	Saint Lawrence	neonate_mortality	2020	NA	15.25
Bel00405	Saint Lawrence	Saint Lawrence	2003	male	30.34
Bel00418	Saint Lawrence	*excluded	2003	male	53.52
Bel00715	Saint Lawrence	Saint Lawrence	2009	female	25.26
Bel00739	Saint Lawrence	Saint Lawrence	2009	female	17.82
Bel00812	Saint Lawrence	Saint Lawrence	2011	male	23.41
Bel01380	Saint Lawrence	Saint Lawrence	2019	female	23.31
Bel01423	Saint Lawrence	Saint Lawrence	2018	female	18.50
Bel01425	Saint Lawrence	Saint Lawrence	2018	NA	21.38
Bel01426	Saint Lawrence	Saint Lawrence	2018	NA	12.76
Bel01559	Saint Lawrence	Saint Lawrence	2021	NA	23.94
HB_Sanikiluaq_26	Sanikiluaq	BIS	2018	male	13.83
HB_Sanikiluaq_27	Sanikiluaq	BIS	2018	female	11.11
HB_Sanikiluaq_28	Sanikiluaq	BIS	2018	female	12.08
HB_Sanikiluaq_29	Sanikiluaq	BIS	2018	female	13.34
HB_Sanikiluaq_30	Sanikiluaq	BIS	2018	female	13.56
HB_Sanikiluaq_31	Sanikiluaq	BIS	2019	male	10.98
HB_Sanikiluaq_36	Sanikiluaq	BIS	2018	male	12.51
HB_Sanikiluaq_37	Sanikiluaq	BIS	2018	male	13.10
HB_Sanikiluaq_38	Sanikiluaq	BIS	2019	male	13.01
HB_Sanikiluaq_39	Sanikiluaq	BIS	2019	female	11.62

Table S1

Sample	Population	Sum_ROH	Number_ROH	Mean_ROH	Max_ROH	FROH	FHOM	max_tMRCA	mean_tMRCA
EHA_Cunningham_Inlet_107	EHA	434336186	1726	251643	4241750	0.18	-0.02	377.20	6358.21
EHA_Cunningham_Inlet_63	EHA	410747715	1617	254018	5140559	0.17	-0.07	311.25	6298.76
EHA_Cunningham_Inlet_111	EHA	436180643	1731	251982	3807083	0.19	-0.06	420.27	6349.66
EHA_Cunningham_Inlet_109	EHA	444315796	1744	254768	2937579	0.19	-0.02	544.67	6280.22
EHA_Cunningham_Inlet_110	EHA	437190862	1733	252274	5720221	0.19	-0.06	279.71	6342.31
EHA_Cunningham_Inlet_108	EHA	445520031	1748	254874	3817256	0.19	-0.06	419.15	6277.61
EHA_Cunningham_Inlet_106	EHA	433153591	1663	260465	2996668	0.18	-0.03	533.93	6142.86
EHA_Cunningham_Inlet_101	EHA	449264171	1708	263035	7192459	0.19	-0.02	222.46	6082.84
EHA_Cunningham_Inlet_105	EHA	448910499	1748	256814	4779144	0.19	-0.02	334.79	6230.19
EHA_Cunningham_Inlet_65	EHA	542409077	1745	310836	10808164	0.23	-0.01	148.04	5147.41
HB_Sanikiluaq_39	BIS	412761550	1764	233992	2161555	0.18	-0.03	740.21	6837.85
HB_Sanikiluaq_36	BIS	391493829	1673	234007	2794374	0.17	-0.08	572.58	6837.40
HB_Sanikiluaq_30	BIS	394210532	1719	229326	2905446	0.17	-0.05	550.69	6976.98
HB_Sanikiluaq_38	BIS	408592609	1728	236454	2956155	0.17	-0.08	541.24	6766.64
HB_Sanikiluaq_28	BIS	404703873	1728	234204	5093229	0.17	-0.03	314.14	6831.66
HB_Sanikiluaq_26	BIS	404767130	1766	229200	2421888	0.17	-0.08	660.64	6980.80
HB_Sanikiluaq_27	BIS	403293726	1665	242219	2036438	0.17	-0.04	785.69	6605.61
HB_Sanikiluaq_31	BIS	402004830	1687	238296	2993440	0.17	-0.07	534.50	6714.35
HB_Sanikiluaq_29	BIS	417873768	1728	241825	3192618	0.18	-0.03	501.16	6616.35
HB_Sanikiluaq_37	BIS	418032200	1689	247503	5522887	0.18	-0.06	289.70	6464.57
Bel01348	NVK	390596011	1726	226301	3541030	0.17	-0.10	451.85	7070.22
Bel01342	NVK	385743995	1711	225449	1894556	0.16	-0.10	844.53	7096.94
Bel01346	NVK	391359626	1694	231027	2398523	0.17	-0.10	667.08	6925.60
Bel01350	NVK	378296190	1636	231232	3208723	0.16	-0.10	498.64	6919.45
Bel01351	NVK	400996863	1725	232462	3550054	0.17	-0.04	450.70	6882.85
Bel01349	NVK	410756973	1728	237707	4069861	0.17	-0.09	393.13	6730.99
Bel01345	NVK	414326217	1781	232637	2189665	0.18	-0.05	730.71	6877.67
Bel01343	NVK	401483317	1724	232879	3159963	0.17	-0.05	506.34	6870.52
Bel01347	NVK	405156860	1781	227488	5284847	0.17	-0.05	302.75	7033.33
Bel01344	NVK	429446310	1822	235701	3395170	0.18	-0.04	471.26	6788.28
Bel01425	SLE	857953519	2489	344698	4171523	0.36	0.17	383.55	4641.74
Bel00739	SLE	912488190	2556	356999	3876602	0.39	0.15	412.73	4481.81
Bel00812	SLE	901891448	2534	355916	7805382	0.38	0.15	204.99	4495.44
Bel00715	SLE	873717331	2492	350609	8954430	0.37	0.14	178.68	4563.49
Bel01380	SLE	875193458	2449	357368	5133287	0.37	0.14	311.69	4477.18
Bel01559	SLE	905477573	2477	365554	5099418	0.38	0.19	313.76	4376.92
Bel00405	SLE	915036168	2539	360392	5285718	0.39	0.16	302.70	4439.61
Bel01426	SLE	892508751	2439	365932	8413488	0.38	0.15	190.17	4372.39
Bel01423	SLE	928195852	2461	377162	6077197	0.39	0.21	263.28	4242.21

Table S2

Sample	Population	Sum_ROH	Number_ROH	Mean_ROH	Max_ROH	FROH	FHOM
Bel00739	Adult	793075008	3837	206691	3543914	0.34	-0.04
Bel01423	Adult	818041659	3738	218845	4569462	0.36	0.01
Bel01425	Adult	742596346	3708	200269	4169958	0.32	-0.03
Bel01426	Adult	783460037	3689	212377	4035132	0.34	-0.04
Bel00405	Adult	802762789	3796	211476	3419332	0.35	-0.03
Bel00715	Adult	761572791	3664	207853	8632921	0.33	-0.06
Bel00812	Adult	784109305	3811	205749	4155357	0.34	-0.04
Bel01380	Adult	761085939	3620	210245	4779016	0.33	-0.06
Bel01559	Adult	781787686	3687	212039	4197567	0.34	-0.01
Bel00098	Neonate	808779402	3616	223667	3967359	0.35	-0.03
Bel00126	Neonate	790786742	3678	215005	4260196	0.34	0.00
Bel01015	Neonate	760109761	3681	206496	5042325	0.33	-0.06
Bel00992	Neonate	792782988	3732	212429	4592424	0.34	-0.04
Bel00096	Neonate	788129403	3756	209832	4637629	0.34	0.00
Bel01530	Neonate	783191428	3746	209074	5325913	0.34	0.00
Bel00584	Neonate	785118288	3663	214338	3463896	0.34	-0.05
Bel00990	Neonate	788026416	3750	210140	3030931	0.34	0.00

Table S3
